# Hyperbolic Prototype-Residual Autoencoder for Interpretable Generative Latent Organization

**DOI:** 10.3390/s26154957

**Published:** 2026-08-05

**Authors:** Hyunjong Lee, Jeongho Kwak

**Affiliations:** 1 Department of Electrical Engineering and Computer Science, Daegu Gyeongbuk Institute of Science & Technology (DGIST), Daegu 42988, Republic of Korea; hjlee@dgist.ac.kr; 2 Department of Computer Science and Engineering, Korea University, Seoul 02841, Republic of Korea

**Keywords:** hyperbolic latent space, prototype learning, Poincaré ball, interpretable generative modeling, prototype-guided generation, autoencoder

## Abstract

Interpretable organization of latent spaces remains a central challenge in generative modeling. Most generative models rely on continuous latent variables, but the learned space often lacks an explicit structure that explains how samples are organized or how semantic variation can be controlled. This paper proposes a hyperbolic prototype-residual autoencoder that organizes generative latent representations using a fixed prototype tree embedded in the Poincaré ball. Each encoded sample is assigned to a prototype by hyperbolic distance, and the decoder reconstructs or generates images from a prototype-residual representation. The prototype serves as a semantic anchor, while the residual captures local instance-level variation around the selected prototype. The framework combines prototype semantic learning, MMD-based latent spreading, and structural regularizers to align encoder–decoder behavior with the predefined hyperbolic hierarchy. Prototype semantic learning encourages fixed prototypes to decode into representative visual anchors, while MMD encourages encoded samples to occupy broad regions of the hyperbolic latent space. Experiments on MNIST and CelebA show interpretable coarse-to-fine behavior through radial decoding and prototype decoding, suggesting that fixed hyperbolic prototype trees provide an effective scaffold for prototype-guided image reconstruction and generation.

## 1. Introduction

Autoencoder-based generative models learn compact latent representations by encoding high-dimensional observations into a lower-dimensional space and reconstructing them through a decoder. Variants such as variational autoencoders (VAEs) [1], Wasserstein autoencoders (WAEs) [2], and vector-quantized variational autoencoders (VQ-VAEs) [3] have advanced this framework by introducing probabilistic regularization, distribution matching, or discrete codebooks. Despite these developments, the organization of the latent space remains a central challenge for both generative modeling and representation learning.

Most autoencoder-based models assume a Euclidean latent space. While computationally convenient, Euclidean latent spaces provide a flat geometric structure and offer limited geometric inductive bias for representing hierarchical or coarse-to-fine semantic variation. Consequently, reconstruction objectives may preserve sample-specific information without imposing a coherent global organization on the latent space. In particular, when semantic factors are organized across different levels of abstraction, a flat Euclidean space may not provide a suitable inductive bias for representing hierarchical relations efficiently [4,5].

Hyperbolic geometry offers a natural mathematical alternative for modeling hierarchical structures. Characterized by negative curvature and exponential volume growth, hyperbolic space can represent tree-like relationships more compactly than Euclidean space. This geometric property has motivated several hyperbolic autoencoding models [4], including hyperbolic VAEs, Poincaré VAEs [5], hyperbolic text-generation VAEs [6], and hyperbolic vector-quantized autoencoders [7].

However, existing hyperbolic autoencoding models typically introduce hierarchy implicitly through the choice of latent geometry itself, either by defining hyperbolic priors and posteriors or by placing codebooks in negatively curved space. In domains such as image generation, training data usually do not provide explicit hierarchical labels or tree-structured supervision. Therefore, merely adopting a hyperbolic latent space does not guarantee the emergence of a semantically interpretable hierarchy. A central question is how to construct an explicit, data-driven hierarchy in a hyperbolic latent space while preserving the continuous variation required for reconstruction.

To address this question, we propose a hyperbolic prototype-residual autoencoder. Instead of relying solely on the implicit structural bias of hyperbolic geometry, we impose an explicit hierarchical prototype tree within the Poincaré ball. Each prototype acts as a semantic anchor, whose target is constructed from the subset of data assigned to its corresponding region. Through this mechanism, hierarchical semantic levels are induced via prototype-wise data aggregation rather than assumed to exist a priori.

Given an input image, the encoder maps it to a continuous point in the Poincaré ball. The nearest leaf prototype is selected as the decoding anchor, and the residual between the encoded point and the selected prototype is used to preserve sample-specific variation. Thus, the prototype captures coarse semantic structure, while the residual retains local information required for reconstruction. To further encourage the encoded representations to occupy broad regions of the hyperbolic latent space rather than collapsing into narrow regions, we regularize the aggregated latent distribution using a Maximum Mean Discrepancy (MMD) objective [8]. In our implementation, MMD is computed after mapping both encoded samples and target samples to the tangent space at the origin.

The main contributions of this work are summarized as follows:

- We propose a hyperbolic prototype-residual autoencoder that combines continuous latent encoding with an explicit fixed prototype hierarchy in the Poincaré ball.
- The model establishes a data-driven semantic hierarchy by assigning representations to hyperbolic prototypes and optimizing prototype decodings toward aggregated semantic targets.
- By decomposing generation into prototype-level semantic anchoring and continuous residual variation, the proposed framework enables hierarchical organization without fully discretizing the latent representation.
- An MMD-based distribution matching objective regularizes the aggregated latent distribution and encourages broad coverage of the hyperbolic latent space.

## 2. Related Work

### 2.1. Autoencoder-Based Generative Models

Autoencoders constitute one of the most fundamental frameworks for learning compact latent representations from high-dimensional data. A standard autoencoder consists of an encoder that maps an input sample *x* to a latent representation *z*, and a decoder that reconstructs the input from this latent code. The model is typically trained by minimizing a reconstruction objective, such as the mean squared error between the original input and its reconstruction. Early deep autoencoders demonstrated that multilayer neural networks can learn nonlinear low-dimensional codes that are more flexible than linear dimensionality reduction methods such as principal component analysis [9]. In this sense, autoencoders provide a natural starting point for representation learning since they encourage the latent space to retain information that is sufficient for reconstructing the observed data.

Beyond simple dimensionality reduction, autoencoders have also been studied as unsupervised representation learning models. Denoising autoencoders, for example, train the model to reconstruct clean inputs from corrupted observations, thereby encouraging the latent representation to capture stable and robust factors of variation rather than simply copying pixel-level details [10,11,12,13,14,15,16,17]. Building upon this foundation, the concept of denoising has recently catalyzed a paradigm shift in generative modeling. Denoising Diffusion Probabilistic Models (DDPMs) [15] formalize this by defining a Markov chain of denoising autoencoders trained to reverse a gradual noising process. By iteratively refining a highly corrupted latent state back into a clean observation, DDPMs demonstrate that sequential denoising not only learns robust representations but also enables the generation of remarkably high-fidelity samples.

This idea is closely related to the manifold-learning interpretation of autoencoders: if natural data concentrate near a lower-dimensional manifold embedded in the ambient observation space, then reconstruction-based learning can be viewed as a way of learning coordinates or features associated with that manifold. As discussed in broader surveys on representation learning, autoencoders are therefore not only compression models, but also mechanisms for discovering useful explanatory factors in data [18,19,20,21].

However, the basic autoencoder objective alone does not fully specify the global structure of the latent space. Since reconstruction loss primarily constrains the decoder on latent codes produced by the encoder, the resulting latent space may be irregular, discontinuous, or poorly organized for sampling and generation. This limitation motivated probabilistic and distribution-matching extensions of autoencoders. VAEs [1] introduce a probabilistic latent variable model by learning an approximate posterior distribution and regularizing it toward a prior distribution, typically through the Kullback–Leibler divergence. This formulation improves the generative interpretation of autoencoders by imposing a continuous latent distribution from which new samples can be drawn. Nevertheless, the commonly used Euclidean Gaussian prior may be insufficient when the data possess non-Euclidean, hierarchical, or multi-scale structure.

WAEs [2] provide another important extension by replacing the standard variational regularization with a distribution-matching objective between the aggregated posterior and a chosen prior distribution. Rather than enforcing each individual posterior to match the prior, WAE-style models regularize the overall encoded data distribution. This perspective is particularly relevant to models that seek to control the geometry or coverage of the latent space, because the latent distribution can be shaped through divergence or discrepancy measures such as MMD. In this respect, WAEs establish an important connection between reconstruction-based learning and global latent-space regularization.

Latent distribution matching can be implemented through several different objectives. VAEs regularize each approximate posterior toward a prior using the Kullback–Leibler divergence [1], whereas adversarial autoencoders use a discriminator to match the aggregated posterior to a target prior [22]. WAEs derive their objective from an optimal-transport formulation and relax aggregated-posterior matching using discrepancy measures such as adversarial divergence or MMD [2]. Kernel-based MMD compares distributions through their mean embeddings in a reproducing kernel Hilbert space [8]. In the proposed framework, we adopt MMD because it provides a direct, non-adversarial, and sample-based objective for matching the aggregated encoded distribution to the hyperbolic target distribution. Specifically, both distributions are mapped to the tangent space at the origin and compared using a multi-kernel RBF discrepancy. Thus, MMD is used as a tractable latent-spreading regularizer, without claiming that it is universally superior to alternative distribution-matching objectives.

A different line of work introduces discreteness into the latent space. VQ-VAE [3] replaces continuous latent variables with discrete codebook entries selected by nearest-neighbor quantization. This design allows the model to learn a finite set of reusable latent codes and has been influential in image, audio, and generative modeling. The vector-quantization mechanism can be interpreted as assigning each encoded representation to a prototype-like code. However, in standard VQ-VAE, the selected code typically replaces the continuous encoder output for decoding, and the codebook is learned directly from data without an explicit geometric hierarchy.

To overcome the limited expressivity of such single quantization, the Residual-Quantized VAE (RQ-VAE) [23] was introduced. RQ-VAE iteratively quantizes the residual between the continuous feature vector and the quantized code, thereby constructing an implicit coarse-to-fine hierarchy. While the use of residuals significantly improves representation precision, this framework inherently remains dependent on discrete codebooks. Consequently, its latent representation remains based primarily on discrete codebook compositions rather than on an explicitly continuous geometric manifold for organizing semantic variation.

From a prototype-learning perspective, vector-quantized generative models use learned codebook vectors as reusable discrete latent representatives [3,23]. VQ-VAE maps each encoded representation to a learned codebook entry through nearest-neighbor vector quantization [3], whereas RQ-VAE recursively quantizes the remaining residual through successive quantization stages, representing each feature as a stack of discrete codes [23]. These codebook representations can be viewed as prototype-like, but they do not explicitly impose a fixed geometric hierarchy over the latent representatives. In contrast, our prototypes are fixed hierarchical anchors embedded in the Poincaré ball. They are explicitly trained to decode into representative images through prototype semantic learning, while a continuous coordinate residual is retained for instance-level reconstruction and generation.

These autoencoder variants show that latent-space design is central to generative modeling. Standard autoencoders focus on reconstruction, VAEs introduce probabilistic latent regularization, WAEs emphasize aggregated distribution matching, while VQ-VAEs and RQ-VAEs organize representations through discrete codebooks. Our method builds on this autoencoding perspective but differs from these approaches in two key respects. First, the latent representation remains continuous rather than being fully replaced by a discrete code. Second, the latent space is structured by a fixed hierarchical prototype tree in the Poincaré ball. The nearest prototype is used as a semantic anchor, while the residual between the encoded point and the selected prototype preserves local variation for reconstruction. Thus, our model can be viewed as a prototype-residual autoencoder that combines reconstruction-based learning with explicit hierarchical organization of the latent space.

### 2.2. Hyperbolic Latent Autoencoders

While the autoencoder-based models discussed above provide powerful frameworks for latent representation learning, most of them assume that the latent space is Euclidean. This assumption is mathematically convenient, since Euclidean spaces support simple Gaussian priors, vector arithmetic, and standard neural network operations. However, Euclidean latent spaces may be inefficient for representing data with inherently hierarchical or tree-like structure. In particular, when semantic relations are organized across multiple levels of abstraction, a flat Euclidean latent space may require a large number of dimensions to preserve hierarchical distances and neighborhood relations [24,25]. Hyperbolic geometry has therefore been introduced into latent-variable models as a way to provide a stronger inductive bias for hierarchical representation learning [4,5,26,27,28].

Nagano et al. [4] proposed a Gaussian-like probability distribution on hyperbolic space, often referred to as a pseudo-hyperbolic Gaussian or wrapped hyperbolic distribution. By defining probabilistic latent variables directly on hyperbolic space, their framework enables a hyperbolic analogue of the VAE. This line of work is important because it addresses a core technical difficulty in non-Euclidean generative modeling: VAEs requires tractable sampling, density evaluation, and reparameterization-like operations, all of which are nontrivial on curved manifolds. Hyperbolic VAEs therefore extend the probabilistic autoencoding framework by replacing the Euclidean latent distribution with a distribution adapted to negatively curved geometry.

Poincaré variational autoencoders further develop this idea by using the Poincaré ball model as the latent space of VAEs [5]. The motivation is that many real-world datasets exhibit hierarchical organization, whereas conventional VAEs typically map data into Euclidean latent spaces. Since hyperbolic space expands exponentially with radius, it can represent tree-like structures more compactly than Euclidean space. In this setting, points near the origin may represent more general or abstract factors, while points farther from the origin can encode more specific information. Poincaré VAEs thus provide a natural geometric framework for learning continuous hierarchical representations within a generative model.

Another related model is APo-VAE [28], which investigates text generation in hyperbolic latent space. Natural language often contains hierarchical structure arising from syntax, semantics, and discourse organization. APo-VAE addresses this by defining both the prior and the variational posterior over a Poincaré ball using wrapped normal distributions. In addition, it introduces an adversarial training procedure based on a primal-dual formulation of the KL divergence to improve training stability. This work demonstrates that hyperbolic latent spaces can be useful not only for representation learning, but also for generative tasks such as language modeling and dialog-response generation.

More recently, hyperbolic geometry has also been combined with vector-quantized autoencoders. HVQ-VAE extends VQ-VAE by learning latent embeddings and codebook vectors in hyperbolic space rather than in Euclidean space [7]. This approach is motivated by the observation that discrete codebooks in standard VQ-VAE do not explicitly encode hierarchical relations among codes. By introducing a hyperbolic geometric prior into the quantized latent space, HVQ-VAE aims to improve the representational capacity of the codebook and to better capture hierarchical structure. In this sense, HVQ-VAE lies at the intersection of hyperbolic latent modeling and discrete codebook-based autoencoding.

Taken together, prior hyperbolic autoencoding models show that negatively curved latent spaces can improve the modeling of hierarchical structure. However, most existing approaches introduce hyperbolic geometry through the choice of latent prior, posterior, or codebook. Our method follows a different direction. Rather than learning a hyperbolic distribution or a hyperbolic codebook alone, it imposes an explicit prototype hierarchy in the Poincaré ball. The selected prototype provides a semantic decoding anchor, while the residual from that prototype retains continuous local variation. This design allows the latent space to combine hierarchical organization with reconstruction-oriented flexibility.

## 3. Mathematical Background and Preliminaries

In this section, we briefly review the mathematical foundations underlying our proposed generative model. We begin by introducing the fundamentals of hyperbolic geometry, with a specific focus on the Poincaré ball model that serves as our structured latent space. Subsequently, we outline the principles of optimal transport and distribution matching—specifically the Wasserstein distance and Maximum Mean Discrepancy (MMD)—which are essential for the global regularization of the latent distribution.

### 3.1. Riemannian Manifolds and the Poincaré Ball

A Riemannian manifold is a smooth manifold M equipped with a Riemannian metric *g*, which defines an inner product on the tangent space $T_{z}M$ at each point z∈M. This metric allows geometric quantities such as distances, angles, and volumes to be defined. Hyperbolic space is a Riemannian manifold with constant negative curvature −c, where c>0.

Among several equivalent models of hyperbolic space, we adopt the Poincaré ball model because of its conformal property, which means that it preserves angles locally with respect to the Euclidean metric. This property makes the model particularly convenient for geometric visualization and neural representation learning. The *d*-dimensional Poincaré ball with curvature −c is defined as

$$B_{c}^{d}=\{z\in R^{d}{:c∥z∥}_{2}^{2}<1\}.$$

The Riemannian metric tensor $g_{z}^{c}$ of the Poincaré ball is conformal to the Euclidean metric $g^{E}$ and is given by

(1) $$g_{z}^{c}={\left(\frac{2}{1-{c∥z∥}_{2}^{2}}\right)}^{2}g^{E}.$$

Under this metric, Euclidean displacements near the boundary of the ball correspond to increasingly large hyperbolic distances. A fundamental property of hyperbolic space is its exponential volume growth with respect to the geodesic radius, which makes it well suited for representing hierarchical and tree-like structures. The hyperbolic distance induced between two points $z,y\in B_{c}^{d}$ is

(2) $$d_{B_{c}}\left(z,y\right)=\frac{1}{\sqrt{c}}\mathrm{arcosh}\left(1+2c\frac{{∥z-y∥}_{2}^{2}}{{(1-c∥z∥}_{2}^{2})(1-{c∥y∥}_{2}^{2})}\right).$$

A particularly important case is the radial distance from the origin 0 to a point *z*, which simplifies to

(3) $$d_{B_{c}}\left(0,z\right)=\frac{2}{\sqrt{c}}\mathrm{artanh}\left(\sqrt{c}{∥z∥}_{2}\right).$$

This relation shows that moving a point toward the boundary corresponds to an increasingly large hyperbolic radius. It provides a useful geometric interpretation of the radial position in the Poincaré ball, especially in settings where radial depth is associated with hierarchical organization.

### 3.2. Tangent Spaces, Exponential and Logarithmic Maps

Because hyperbolic space is a curved manifold, standard Euclidean vector operations are not intrinsically defined on the manifold itself. To relate Euclidean operations to the manifold geometry, one commonly uses tangent spaces. For a point $z\in B_{c}^{d}$, the tangent space $T_{z}B_{c}^{d}$ is a *d*-dimensional vector space that provides a local first-order approximation of the manifold around *z*. Although $T_{z}B_{c}^{d}$ is isomorphic to $R^{d}$ as a vector space, its geometric interpretation depends on the base point *z* and the associated Riemannian metric.

Mappings between the manifold and its tangent spaces are given by the exponential and logarithmic maps. The exponential map at *z*, denoted by ${\mathrm{Exp}}_{z}^{c}$, maps a tangent vector $v\in T_{z}B_{c}^{d}$ to a point on the manifold by following the geodesic starting from *z* with initial velocity *v*. Conversely, the logarithmic map at *z*, denoted by ${\mathrm{Log}}_{z}^{c}$, maps a point on the manifold back to a tangent vector in $T_{z}B_{c}^{d}$. These maps are formally denoted as

$${\mathrm{Exp}}_{z}^{c}:T_{z}B_{c}^{d}\to B_{c}^{d},\;{\mathrm{Log}}_{z}^{c}:B_{c}^{d}\to T_{z}B_{c}^{d}.$$

A particularly important case is the tangent space at the origin, $T_{0}B_{c}^{d}$, which can be identified with the Euclidean space $R^{d}$. For a nonzero tangent vector $v\in T_{0}B_{c}^{d}$, the exponential map at the origin is given by

(4) $${\mathrm{Exp}}_{0}^{c}\left(v\right)=\tanh(\sqrt{c}{∥v∥}_{2})\frac{v}{\sqrt{c}{∥v∥}_{2}}.$$

Conversely, for a nonzero point $y\in B_{c}^{d}$, the logarithmic map at the origin is given by

(5) $${\mathrm{Log}}_{0}^{c}\left(y\right)=\mathrm{artanh}(\sqrt{c}{∥y∥}_{2})\frac{y}{\sqrt{c}{∥y∥}_{2}}.$$

For v=0 or y=0, the corresponding maps are defined to return 0. These operations provide a fundamental interface between Euclidean tangent spaces and the Poincaré ball.

### 3.3. Wasserstein Distance and Maximum Mean Discrepancy

Wasserstein distance provides a geometric way to measure the discrepancy between two probability distributions. Unlike divergence measures such as the Kullback–Leibler divergence, which compare probability densities pointwise, Wasserstein distance is based on the cost of transporting mass from one distribution to another. For two probability distributions *P* and *Q* defined in a metric space X, the *p*-Wasserstein distance is defined as

(6) $$W_{p}\left(P,Q\right)={\left(\underset{\gamma \in \Pi (P,Q)}{\inf}\int_{X\times X}d{\left(x,y\right)}^{p}\;d\gamma \left(x,y\right)\right)}^{1/p},$$

where Π(P,Q) denotes the set of all couplings whose marginals are *P* and *Q*, and d(x,y) is the ground metric between samples. Intuitively, $W_{p}\left(P,Q\right)$ measures the minimum transportation cost required to transform one distribution into the other.

This optimal-transport perspective has been influential in generative modeling because generation can be interpreted as matching the model distribution to the data distribution. In autoencoder-based models, this idea leads to the Wasserstein autoencoder framework, where reconstruction learning is combined with a regularization term that matches the aggregated posterior distribution to a chosen prior distribution [2]. Rather than enforcing each individual posterior to match the prior, this formulation regularizes the overall encoded distribution. As a result, the latent space can be shaped globally while preserving the reconstruction capability of the autoencoder.

However, directly computing optimal-transport distances can be computationally demanding, especially in high-dimensional neural generative models. For this reason, tractable discrepancy measures are often used to implement distribution matching objectives in practice. One such discrepancy measure is Maximum Mean Discrepancy (MMD), which compares two probability distributions through their mean embeddings in a reproducing kernel Hilbert space (RKHS). Given two distributions *P* and *Q*, MMD is defined as

(7) $$\mathrm{MMD}\left(P,Q;H\right)={∥E_{x∼P}\left[\phi \left(x\right)\right]-E_{y∼Q}\left[\phi \left(y\right)\right]∥}_{H},$$

where ϕ(·) is the feature map associated with a positive definite kernel k(·,·), and H is the corresponding RKHS.

Using the kernel trick, the squared MMD can be written without explicitly computing the feature map:

(8) $$\begin{array}{cc} {\mathrm{MMD}}^{2}\left(P,Q\right)= & E_{x,x^{\prime }∼P}\left[k(x,x^{\prime })\right]+E_{y,y^{\prime }∼Q}\left[k(y,y^{\prime })\right]\\ \; & -2E_{x∼P,y∼Q}\left[k(x,y)\right]. \end{array}$$

For finite samples ${\left\{x_{i}\right\}}_{i=1}^{n}∼P$ and ${\left\{y_{j}\right\}}_{j=1}^{m}∼Q$, this quantity can be estimated empirically as

(9) $$\begin{array}{cc} {\hat{\mathrm{MMD}}}^{2}\left(P,Q\right)= & \frac{1}{n^{2}}\sum_{i=1}^{n}\sum_{i^{\prime }=1}^{n}k\left(x_{i},x_{i^{\prime }}\right)+\frac{1}{m^{2}}\sum_{j=1}^{m}\sum_{j^{\prime }=1}^{m}k\left(y_{j},y_{j^{\prime }}\right)\\ \; & -\frac{2}{nm}\sum_{i=1}^{n}\sum_{j=1}^{m}k\left(x_{i},y_{j}\right). \end{array}$$

A common choice is the radial basis function (RBF) kernel,

(10) $$k_{\sigma }\left(x,y\right)=\exp\left(-\frac{{∥x-y∥}_{2}^{2}}{2\sigma^{2}}\right),$$

where σ>0 is the kernel bandwidth. In practice, multiple bandwidths can be combined to make the discrepancy sensitive to both local and global distributional differences.

(11) $$k\left(x,y\right)=\sum_{\sigma \in \Sigma }\exp\left(-\frac{{∥x-y∥}_{2}^{2}}{2\sigma^{2}}\right).$$

Thus, MMD is not itself a Wasserstein distance, but it serves as a tractable distribution-matching penalty in Wasserstein-style autoencoding frameworks. It allows the aggregated latent distribution to be aligned with a target distribution without requiring explicit likelihood estimation or direct optimal-transport computation.

## 4. Methods

### 4.1. Overview

This section describes the overall methodology of the proposed framework. In this study, we assume the latent space to be hyperbolic and aim to structure the latent representation by integrating a fixed hierarchical prototype tree within this hyperbolic manifold. Specifically, we aim to organize latent representations from coarse semantic anchors to fine-grained instance-specific variations. Under this framework, each input image is embedded into a continuous latent representation, which is then interpreted within a prototype-based hierarchical structure.

The remainder of this section is organized as follows. First, we provide an overview of the overall architecture of the proposed model, followed by a description of the fixed prototype tree constructed in the Poincaré ball and the methodology for prototype arrangement. Next, we detail the hyperbolic embedding process, Maximum Mean Discrepancy (MMD)-based latent spreading, and nearest leaf prototype assignment. Subsequently, we explain prototype semantic learning and prototype-residual decoding. Finally, we conclude by summarizing the total training objective.

**Figure 1:**
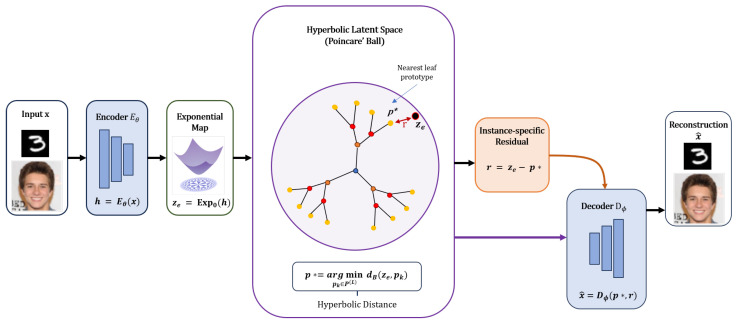
Overall pipeline of the proposed method. The differently colored dots represent prototypes at different hierarchical depths of the fixed prototype tree. The asterisk indicates the nearest leaf prototype $p^{*}$ assigned to the encoded latent point $z_{e}$. The selected prototype $p^{*}$ and the coordinate residual $r=z_{e}-p^{*}$ are jointly provided to the decoder for image reconstruction.

Figure 1 illustrates the overall pipeline of the proposed method. The proposed model maps each input into a hyperbolic latent space and structures the resulting representation using a fixed hierarchical prototype tree. Given an input image *x*, the encoder first produces a tangent-space latent vector *h*, which is mapped to a hyperbolic latent representation $z_{e}$ on the Poincaré ball. The encoded point $z_{e}$ is then assigned to the nearest leaf prototype $p^{*}$ according to the hyperbolic distance.

The selected prototype $p^{*}$ serves as a semantic anchor for the corresponding latent point. To preserve instance-specific details, we compute a residual between the encoded point and the selected prototype and use both components as decoder inputs. The overall reconstruction process is formulated as follows:

(12) $$\begin{array}{cc} h & =E_{\theta }\left(x\right), \end{array}$$

(13) $$\begin{array}{cc} z_{e} & ={\mathrm{Exp}}_{0}^{c}\left(h\right), \end{array}$$

(14) $$\begin{array}{cc} p^{*} & =\arg\min_{p_{k}\in P^{\left(L\right)}}d_{B_{c}}\left(z_{e},p_{k}\right), \end{array}$$

(15) $$\begin{array}{cc} r & =z_{e}-p^{*}, \end{array}$$

(16) $$\begin{array}{cc} \hat{x} & =D_{\phi }\left(p^{*},r\right). \end{array}$$

where $P^{\left(L\right)}$ denotes the set of leaf prototypes and $d_{B_{c}}\left(\cdot ,\cdot \right)$ is the hyperbolic distance in the Poincaré ball with curvature −c. The detailed interpretation of *r* as a coordinate residual is provided in Section 4.5.

### 4.2. Hyperbolic Latent Space and Fixed Prototype Tree

As discussed in Section 4.1, the proposed model embeds the input images into a hyperbolic latent space on the Poincaré ball and structures the latent representations based on a fixed prototype tree. In this section, we first explain the role of the fixed hyperbolic prototype tree and subsequently describe how the prototypes are positioned within the hyperbolic space.

#### 4.2.1. Fixed Hyperbolic Prototype Tree

In the proposed method, prototypes are not learnable code vectors that move freely during the training process; rather, they serve as pre-defined, fixed hierarchical anchors within the hyperbolic latent space, acting as a structural backbone for the data representation. The entire set of prototypes P is defined as the union of prototype subsets across all hierarchical depths ℓ∈{0,1,…,L}:

(17) $$P=\overset{L}{\underset{\ell =0}{⋃}}P^{\left(\ell \right)},\;P^{\left(\ell \right)}={\left\{p_{k}^{\left(\ell \right)}\right\}}_{k=1}^{K_{\ell }},$$

where *L* denotes the maximum depth of the tree, $P^{\left(\ell \right)}$ is the set of prototypes at depth *ℓ*, and $K_{\ell }$ represents the number of prototypes at depth *ℓ*.

Specifically, $P^{\left(0\right)}$ denotes the root prototype, whereas $P^{\left(L\right)}$ represents the set of leaf prototypes. Prototypes at different depths are not treated as independent code vectors; instead, they are organized into a hierarchical tree via parent-child relations. For each depth ℓ≥1, let $\pi_{\ell }\left(k\right)$ denote the parent index of the *k*-th prototype $p_{k}^{\left(\ell \right)}$. The parent of $p_{k}^{\left(\ell \right)}$ is defined as:

(18) $$\mathrm{par}\left(p_{k}^{\left(\ell \right)}\right)=p_{\pi_{\ell }\left(k\right)}^{\left(\ell -1\right)},\;\pi_{\ell }\left(k\right)\in \left\{1,\ldots ,K_{\ell -1}\right\}.$$

This mapping defines the edge structure of the prototype tree by connecting each child prototype to a unique parent prototype at the preceding depth. Accordingly, the edge set E of the entire prototype tree is defined as follows:

$$E=\left\{\left(p_{\pi_{\ell }\left(k\right)}^{\left(\ell -1\right)},\;p_{k}^{\left(\ell \right)}\right)\;|\;\ell =1,\ldots ,L,\;k=1,\ldots ,K_{\ell }\right\}.$$

Furthermore, each leaf prototype $p_{k}^{\left(L\right)}$ is associated with an ancestor path originating from the root by recursively tracing the parent mapping. By letting $a_{L}=k$, the sequence of indices along the path is defined as:

(19) $$a_{\ell -1}=\pi_{\ell }\left(a_{\ell }\right),\;\ell =L,L-1,\ldots ,1.$$

Then, the root-to-leaf path $A(p_{k}^{\left(L\right)})$ is expressed as:

(20) $$A\left(p_{k}^{\left(L\right)}\right)=\left(p_{a_{0}}^{\left(0\right)},p_{a_{1}}^{\left(1\right)},\ldots ,p_{a_{L}}^{\left(L\right)}\right).$$

Such a structure is designed to provide an explicit coarse-to-fine hierarchy within the latent space.

#### 4.2.2. Prototype Placement

For the fixed hyperbolic prototype tree to function as an effective scaffold, the prototypes must be positioned in accordance with the hierarchical expansion of the hyperbolic space rather than being placed arbitrarily. In this study, we arrange the prototypes to progressively expand from the center toward the periphery. Specifically, prototypes at shallower depths represent relatively coarse semantic regions, while child prototypes at deeper levels branch out from their parents to cover more fine-grained domains.

Each prototype at depth *ℓ* is positioned by considering both directional and radial components. Intuitively, the direction of a prototype determines the semantic region it occupies in the latent space, whereas its distance from the origin reflects its depth within the hierarchy. Consequently, higher-level prototypes represent broader semantic areas, while lower-level prototypes branch into multiple directions within the domain of their parent prototype to form finer-grained regions.

To generalize this process, each prototype $p_{k}^{\left(\ell \right)}$ is generated by moving from its parent $p_{\pi_{\ell }\left(k\right)}^{\left(\ell -1\right)}$ along a specified direction $v_{k}^{\left(\ell \right)}$.

This displacement is performed via the exponential map on the Poincaré ball with curvature −c:

(21) $$p_{k}^{\left(\ell \right)}={\mathrm{Exp}}_{p_{\pi_{\ell }\left(k\right)}^{\left(\ell -1\right)}}^{c}\left(s_{\ell }v_{k}^{\left(\ell \right)}\right),\;\ell =1,\ldots ,L,$$

where $s_{\ell }$ denotes the step size at depth *ℓ* that controls the displacement of the child prototype from its parent, and $v_{k}^{\left(\ell \right)}$ is a unit direction vector in the tangent space of the parent prototype.

A crucial aspect of prototype placement is the balance between coverage and overlap. If the coverage of a prototype is too narrow, latent points may fail to stably associate with any specific prototype. Conversely, if the regions are excessively broad or heavily overlapping, prototypes cannot effectively function as distinct semantic anchors. Therefore, we choose the directions and level-wise step sizes to encourage sufficient coverage of the parent’s domain while avoiding excessive overlap.

To achieve this, the placement is designed so that child prototypes maintain hierarchical connectivity by refining their parent’s domain, while simultaneously remaining sufficiently separated from one another. This dual requirement encourages a parent region to be refined into multiple child regions, resulting in a progressively finer hierarchical organization as the depth increases.

In essence, prototypes form a fixed geometric anchor field that serves as a reference for the latent representations as they expand toward the periphery of the Poincaré ball. By providing such a structured framework, prototype placement biases the latent organization toward hierarchical directions and predefined coverage regions rather than leaving it entirely unstructured.

The specific number of prototypes, radial distances per level, and angular spread of child prototypes are selected by considering the trade-off between coverage, uncovered regions, and overlap. The placement score is defined as $S=R_{\mathrm{uncov}}+R_{\mathrm{overlap}}$, where the two terms represent the fractions of shell samples covered by no prototype region and by two or more regions, respectively. Further details are provided in Appendix B. Overall, the proposed placement strategy jointly considers parent–child hierarchy, radial expansion, and angular separation. Consequently, the fixed prototype tree functions as a structural scaffold that induces a coarse-to-fine latent organization within the hyperbolic space.

### 4.3. Data Embedding in Hyperbolic Latent Space

As defined in the previous section, the fixed prototype tree acts as a hierarchical scaffold expanding from the center toward the periphery of the Poincaré ball. This structure aims to organize latent representations from shared coarse anchors to more specific leaf-level regions. Specifically, central or higher-level prototypes are intended to serve as anchors for coarse semantic information shared across multiple samples, whereas peripheral or lower-level prototypes are encouraged to capture more specific and fine-grained variations.

To achieve this, an input image *x* is transformed into a tangent-space representation *h* via an encoder $E_{\theta }$, and then mapped to a hyperbolic latent point $z_{e}$ using the exponential map at the origin:

(22) $$h=E_{\theta }\left(x\right),\;z_{e}={\mathrm{Exp}}_{0}^{c}\left(h\right),\;z_{e}\in B_{c}^{d}.$$

To encourage encoded points to occupy broader radial regions of the Poincaré ball, we employ MMD-based latent spread regularization. In hyperbolic space, the available volume increases exponentially as the geodesic radius grows. Consequently, a hyperbolic volume-aware target distribution assigns more probability mass to regions with larger hyperbolic radii. This regularization encourages the encoded latent distribution to spread toward the periphery, while the fixed prototype tree provides a structural scaffold that biases the latent organization toward predefined hierarchical directions.

For a batch of encoded latent samples ${\left\{z_{i}\right\}}_{i=1}^{B}$, the MMD loss is computed in the tangent space at the origin. Specifically, both encoded latent samples and target samples are mapped by the logarithmic map:

(23) $$u_{i}={\mathrm{Log}}_{0}^{c}\left(z_{i}\right),\;v_{j}={\mathrm{Log}}_{0}^{c}\left({\tilde{z}}_{j}\right),\;{\tilde{z}}_{j}∼p_{B_{c}}.$$

The latent spread regularization is defined as the empirical squared MMD between the two tangent-space sample sets:

(24) $$L_{\mathrm{mmd}}={\hat{\mathrm{MMD}}}^{2}\left({\left\{u_{i}\right\}}_{i=1}^{B},{\left\{v_{j}\right\}}_{j=1}^{B}\right),$$

where ${\hat{\mathrm{MMD}}}^{2}$ is computed using the multi-kernel RBF kernel defined in Equation (11). The target sample ${\tilde{z}}_{j}$ is drawn from the target distribution $p_{B_{c}}$ on the Poincaré ball, and ${\mathrm{Log}}_{0}^{c}\left(\cdot \right)$ maps points on the ball to the tangent space at the origin.

The target distribution is constructed by sampling the angular direction uniformly and drawing the hyperbolic radius ρ from the truncated radial density

(25) $$p\left(\rho \right)=\frac{1}{Z}\exp\left(\alpha \rho \right)\sinh{\left(\sqrt{c}\rho \right)}^{d-1},\;0\leq \rho \leq \rho_{\max}.$$

The constant *Z* normalizes the density, α controls the radial bias toward the periphery, and $\rho_{\max}$ specifies the maximum sampled hyperbolic radius.

By matching encoded samples to this volume-aware target distribution, the MMD regularizer encourages the latent embeddings to occupy broad radial regions of the hyperbolic space. Together with the fixed prototype tree, this promotes an organization in which latent representations extend from shared coarse regions toward more specific prototype regions.

### 4.4. Prototype Semantic Learning

Given the fixed hyperbolic prototype structure, we train each prototype to represent the visual semantics of its associated latent region. A prototype should not merely serve as a geometric anchor; it should also be decoded into a representative image of the samples located in its domain. To this end, we construct a semantic target for each active prototype and optimize the decoder so that the prototype with zero residual reconstructs this target.

The root prototype is treated separately from non-root prototypes. Since the root prototype represents the common coarse representation shared by the samples, its semantic target is defined as the batch mean during training:

(26) $${\bar{x}}_{B}=\frac{1}{B}\sum_{i=1}^{B}x_{i},$$

where *B* denotes the batch size. This avoids applying a directional coverage region to the root prototype, which is located at the origin and, therefore, does not induce a well-defined outward radial half-space.

For non-root prototypes, we define prototype-specific regions using outward geodesic half-spaces. Geometrically, in the Poincaré ball, the boundary of a hyperbolic half-space orthogonal to the radial axis corresponds to a Euclidean sphere in the scaled ball coordinates. For a non-root prototype $p_{k}^{\left(\ell \right)}$, ℓ≥1, let

(27) $$a_{k}^{\left(\ell \right)}=\sqrt{c}{∥p_{k}^{\left(\ell \right)}∥}_{2},\;u_{k}^{\left(\ell \right)}=\frac{\sqrt{c}\;p_{k}^{\left(\ell \right)}}{a_{k}^{\left(\ell \right)}}.$$

The corresponding coverage region $C_{k}^{\left(\ell \right)}$ is defined as

(28) $$C_{k}^{\left(\ell \right)}=\left\{z\in B_{c}^{d}\;|\;{∥\sqrt{c}z-q_{k}^{\left(\ell \right)}∥}_{2}^{2}\leq {\left(R_{k}^{\left(\ell \right)}\right)}^{2}\right\},$$

where $q_{k}^{\left(\ell \right)}$ and $R_{k}^{\left(\ell \right)}$ denote the Euclidean center and radius of the sphere defining the half-space boundary in the scaled coordinates:

(29) $$q_{k}^{\left(\ell \right)}=\frac{1+{\left(a_{k}^{\left(\ell \right)}\right)}^{2}}{2a_{k}^{\left(\ell \right)}}u_{k}^{\left(\ell \right)},\;R_{k}^{\left(\ell \right)}=\frac{1-{\left(a_{k}^{\left(\ell \right)}\right)}^{2}}{2a_{k}^{\left(\ell \right)}}.$$

Only samples whose latent embeddings lie inside $C_{k}^{\left(\ell \right)}$ are used to construct the semantic target of $p_{k}^{\left(\ell \right)}$.

Let

(30) $$A_{k}^{\left(\ell \right)}=\left\{i|z_{i}\in C_{k}^{\left(\ell \right)}\right\}$$

denote the active sample set of prototype $p_{k}^{\left(\ell \right)}$. For active prototypes satisfying $A_{k}^{\left(\ell \right)}\neq \emptyset$, the semantic target is defined as a distance-weighted mean:

(31) $${\bar{x}}_{k}^{\left(\ell \right)}=\frac{\sum_{i\in A_{k}^{\left(\ell \right)}}{\tilde{w}}_{ik}^{\left(\ell \right)}x_{i}}{\sum_{i\in A_{k}^{\left(\ell \right)}}{\tilde{w}}_{ik}^{\left(\ell \right)}},\;\ell \geq 1.$$

The unnormalized weight is defined by exponential decay with respect to the hyperbolic distance:

(32) $${\tilde{w}}_{ik}^{\left(\ell \right)}=\exp\left(-\frac{d_{B_{c}}\left(z_{i},p_{k}^{\left(\ell \right)}\right)}{\tau_{p}}\right),$$

where $\tau_{p}$ is a temperature parameter. Thus, samples closer to the prototype in hyperbolic distance contribute more strongly to the prototype target.

The prototype is decoded with a zero residual,

(33) $${\hat{x}}_{k}^{\left(\ell \right)}=D_{\phi }\left(p_{k}^{\left(\ell \right)},0\right),$$

and the decoder is optimized so that each decoded prototype matches its semantic target. Let

(34) $$I^{\left(\ell \right)}=\left\{k|A_{k}^{\left(\ell \right)}\neq \emptyset \right\}$$

denote the set of active prototypes at depth *ℓ*. The prototype semantic loss is then defined as

(35) $$\begin{array}{cc} L_{\mathrm{proto}}= & {∥D_{\phi }\left(p^{\left(0\right)},0\right)-{\bar{x}}_{B}∥}_{2}^{2}\\ \; & +\sum_{\ell =1}^{L}\sum_{k\in I^{\left(\ell \right)}}{∥D_{\phi }\left(p_{k}^{\left(\ell \right)},0\right)-{\bar{x}}_{k}^{\left(\ell \right)}∥}_{2}^{2}. \end{array}$$

This objective encourages the fixed prototype coordinates to become interpretable by training the decoder to associate each prototype with a representative image of its corresponding region. The root prototype captures the common coarse appearance of the batch, while non-root prototypes are encouraged to represent progressively more specific visual characteristics within their coverage regions.

### 4.5. Prototype-Residual Decoding and Generation

Through Prototype Semantic Learning, fixed prototypes are no longer mere coordinates but are interpreted by the decoder as meaningful representative images. That is, $D_{\phi }\left(p,0\right)$ represents the prototype-level semantic information of the region associated with prototype *p*. This section describes how to combine this prototype-level semantic anchor with residual information for image reconstruction and prototype-guided synthesis.

Once an input image *x* is embedded into $z_{e}$ via the encoder and the nearest leaf prototype $p^{*}\left(z_{e}\right)$ is selected, we define the residual between $z_{e}$ and $p^{*}\left(z_{e}\right)$ as follows:

(36) $$r=z_{e}-p^{*}\left(z_{e}\right).$$

Although prototype assignment relies on the hyperbolic distance, the residual is represented as a coordinate offset in the ambient coordinate representation of the Poincaré ball. Since the selected prototype is determined from $z_{e}$ and $z_{e}=p^{*}\left(z_{e}\right)+r$, the pair $\left(p^{*}\left(z_{e}\right),r\right)$ preserves the information contained in the original latent representation. Accordingly, this construction should not be interpreted as information-theoretic disentanglement or statistical independence between the prototype and residual. Instead, it provides a decoder-side decomposition with complementary roles: the prototype identifies and conditions the selected hierarchical region, whereas the residual specifies variation relative to that anchor. Therefore, *r* is not interpreted as an intrinsic hyperbolic displacement, but as a decoder-side coordinate residual relative to the selected prototype.

The decoder takes both the selected prototype and the residual as inputs to reconstruct the image:

(37) $$\hat{x}=D_{\phi }\left(p^{*}\left(z_{e}\right),r\right).$$

In this architecture, $p^{*}\left(z_{e}\right)$ provides the prototype-level semantic anchor to which the sample belongs, and *r* captures the instance-specific variation within that prototype’s domain. Therefore, instead of simply transforming a single latent vector into an image, the decoder reconstructs the image by combining the prototype-level semantic anchor with the fine-grained variations provided by the residual.

Architecturally, the decoder adopts a forward(prototype, residual) input structure, where the prototype is designed to act as a semantic condition and the residual as the within-prototype variation. More specifically, the decoder transforms the prototype and the residual into separate features before fusing them. The prototype feature serves as a conditioning signal that modulates the residual feature. Consequently, the same residual vector can lead to different reconstructions depending on the prototype condition. Conversely, varying the residual for a given prototype allows for the generation of diverse instance-level variations within that prototype’s semantic region. In our implementation, the decoder explicitly separates the prototype and residual branches, applying modulation parameters—generated from the prototype feature—to the residual feature.

This prototype-residual decomposition supports both reconstruction and prototype-guided generation. In reconstruction, the encoded point is decomposed into its nearest prototype and coordinate residual, and the image is restored through $D_{\phi }\left(p^{*},r\right)$.

For random prototype-guided generation, a leaf prototype *p* is selected uniformly from the complete set of leaf prototypes $P^{\left(L\right)}$, without occupancy weighting or manual selection. Conditional on the selected prototype, the angular coordinates and the radial coordinate are sampled independently from uniform distributions over the admissible ranges defining its coverage region $C_{p}$. This procedure yields a latent point

(38) $$p∼\mathrm{Unif}\;\left(P^{\left(L\right)}\right),\;z_{s}\in C_{p}.$$

The corresponding coordinate residual is then defined as

(39) $$r_{s}=z_{s}-p.$$

Prototype-guided generation is formulated as

(40) $$x_{\mathrm{proto}}=D_{\phi }\left(p,0\right),\;x_{\mathrm{gen}}=D_{\phi }\left(p,r_{s}\right).$$

The term $D_{\phi }\left(p,0\right)$ generates the representative image associated with the prototype itself, while $D_{\phi }\left(p,r_{s}\right)$ synthesizes an image corresponding to a sampled latent variation within the coverage region of the selected prototype. This procedure does not require learning an explicit residual prior; the residual is induced by the coordinate offset between the sampled latent point and the selected prototype.

From this perspective, generation in the proposed method is not an unconditional random latent sampling process. Rather, it is guided by selecting a semantic anchor from the fixed hyperbolic prototype tree and using residual variation around that anchor. In essence, the prototype tree provides the global semantic structure for generation, whereas the residual contributes local instance-level variation around the selected prototype. This architecture enables a prototype-level interpretation of the generated images and provides a useful way to analyze the types of variation that can appear around a specific leaf prototype.

Conceptually, the proposed decoder can be summarized as

(41) $$\begin{array}{cc} \mathrm{Reconstruction} & \approx \mathrm{Prototype}\;\mathrm{Semantics}\\ \; & \;+\mathrm{Residual}\;\mathrm{Variation}. \end{array}$$

Through this decomposition, the model preserves the hierarchical semantic structure provided by the fixed hyperbolic prototype tree while capturing the fine-grained differences of individual images via the residual.

### 4.6. Auxiliary Critic for Image-Level Refinement

Although the proposed prototype-residual autoencoder is primarily designed to organize the latent space through a hyperbolic prototype hierarchy, pixel-wise reconstruction losses often lead to overly smooth output. This effect is particularly noticeable in image regions that contain high-frequency details such as edges, textures, and fine facial structures. To mitigate this issue, we employ an auxiliary critic during training as an image-level refinement module.

The critic is not intended to define or supervise the hyperbolic hierarchy. The prototype tree, prototype assignment, prototype semantic targets, and MMD regularization remain the main mechanisms for latent-space organization. Instead, the critic provides an additional image-level training signal that encourages the model-generated images to retain sharper visual details. Thus, it primarily serves as a complementary refinement component and does not alter the prototype-residual decoding mechanism.

Let $C_{\psi }\left(x\right)$ denote the critic logit for an image *x*, and let BCE denote binary cross-entropy with logits. For an input image $x∼p_{\mathrm{data}}$, the encoder first produces a hyperbolic latent point

(42) $$z_{e}\left(x\right)={\mathrm{Exp}}_{0}^{c}\left(E_{\theta }\left(x\right)\right).$$

The nearest leaf prototype is then selected as $p^{*}\left(z_{e}\left(x\right)\right)$, and the corresponding coordinate residual is computed as

(43) $$r\left(x\right)=z_{e}\left(x\right)-p^{*}\left(z_{e}\left(x\right)\right).$$

The reconstructed image is therefore given by

(44) $${\hat{x}}_{\mathrm{rec}}\left(x\right)=D_{\phi }\left(p^{*}\left(z_{e}\left(x\right)\right),r\left(x\right)\right).$$

In addition to reconstructed images, we also use prototype-region samples as fake images for the critic. For a selected leaf prototype *p*, we randomly sample a latent point $z_{s}$ within its coverage region $C_{p}$ and define the corresponding residual as

(45) $$r_{s}=z_{s}-p,\;z_{s}\in C_{p}.$$

The corresponding coverage-sampled image is then generated by

(46) $${\hat{x}}_{\mathrm{cov}}=D_{\phi }\left(p,r_{s}\right).$$

This sample provides an additional fake image source for the critic, allowing the refinement signal to act not only on reconstructions but also on images generated from local regions around leaf prototypes.

Using both reconstructed and coverage-sampled images, the critic is trained to distinguish real images from model-generated images:

(47) $$\begin{array}{cc} L_{C}= & E_{x∼p_{\mathrm{data}}}\left[\mathrm{BCE}\left(C_{\psi }\left(x\right),1\right)\right]\\ \; & +\omega_{\mathrm{rec}}E_{x∼p_{\mathrm{data}}}\left[\mathrm{BCE}\left(C_{\psi }\left({\hat{x}}_{\mathrm{rec}}\left(x\right)\right),0\right)\right]\\ \; & +\omega_{\mathrm{cov}}E_{{\hat{x}}_{\mathrm{cov}}}\left[\mathrm{BCE}\left(C_{\psi }\left({\hat{x}}_{\mathrm{cov}}\right),0\right)\right], \end{array}$$

where $\omega_{\mathrm{rec}}$ and $\omega_{\mathrm{cov}}$ control the relative contribution of reconstruction-based and coverage-sampled fake images. The autoencoder receives the corresponding refinement signal by encouraging both types of model-generated images to be classified as real:

(48) $$\begin{array}{cc} L_{\mathrm{ref}}= & \omega_{\mathrm{rec}}E_{x∼p_{\mathrm{data}}}\left[\mathrm{BCE}\left(C_{\psi }\left({\hat{x}}_{\mathrm{rec}}\left(x\right)\right),1\right)\right]\\ \; & +\omega_{\mathrm{cov}}E_{{\hat{x}}_{\mathrm{cov}}}\left[\mathrm{BCE}\left(C_{\psi }\left({\hat{x}}_{\mathrm{cov}}\right),1\right)\right]. \end{array}$$

This objective encourages the autoencoder to produce both reconstructed and prototype-region sampled images that are classified as real by the critic, thereby providing an auxiliary image-level refinement signal. Training alternates between critic and autoencoder updates. Generated images are detached during critic updates, whereas the critic parameters are frozen during autoencoder updates. Thus, the critic does not directly define the prototype hierarchy, although its refinement gradients may indirectly influence the learned latent representations.

### 4.7. Training Objective

The proposed model is optimized using three main structural objectives: reconstruction, prototype semantic learning, and latent spread regularization, together with two auxiliary structural regularizers: coverage pullback and parent–child consistency. In addition, the auxiliary critic provides an image-level refinement loss during training. For an input image $x_{i}$, the encoder produces a hyperbolic latent embedding $z_{i}$, which is assigned to the nearest leaf prototype $p_{i}^{*}=p^{*}\left(z_{i}\right)$. The residual is defined as $r_{i}=z_{i}-p_{i}^{*}$, and the reconstruction is given by

(49) $${\hat{x}}_{i}=D_{\phi }\left(p_{i}^{*},r_{i}\right).$$

The reconstruction loss is computed over a batch of size *B*:

(50) $$L_{\mathrm{rec}}=\frac{1}{B}\sum_{i=1}^{B}{∥x_{i}-{\hat{x}}_{i}∥}_{2}^{2}.$$

To assign semantic meaning to the fixed prototypes, we use the prototype semantic loss:

(51) $$\begin{array}{cc} L_{\mathrm{proto}}= & {∥D_{\phi }\left(p^{\left(0\right)},0\right)-{\bar{x}}_{B}∥}_{2}^{2}\\ \; & +\sum_{\ell =1}^{L}\sum_{k\in I^{\left(\ell \right)}}{∥D_{\phi }\left(p_{k}^{\left(\ell \right)},0\right)-{\bar{x}}_{k}^{\left(\ell \right)}∥}_{2}^{2}. \end{array}$$

where ${\bar{x}}_{B}=\frac{1}{B}\sum_{i=1}^{B}x_{i}$ denotes the batch mean image, ${\bar{x}}_{k}^{\left(\ell \right)}$ denotes the semantic target of prototype $p_{k}^{\left(\ell \right)}$, and $I^{\left(\ell \right)}$ is the set of active prototypes at depth *ℓ*.

The latent spread regularization term $L_{\mathrm{mmd}}$, defined in Equation (24), matches the encoded latent distribution to the hyperbolic volume-aware target distribution.

To improve the association between latent embeddings and predefined leaf coverage regions, we use a coverage pullback loss. For each latent point $z_{i}$, let

$$k_{i}^{*}=\arg\min_{k}d_{B_{c}}\left(z_{i},p_{k}^{\left(L\right)}\right)$$

denote the index of the nearest leaf prototype. The coverage pullback loss is defined conceptually as

(52) $$L_{\mathrm{cov}}=\frac{1}{B}\sum_{i=1}^{B}1\left[z_{i}\notin \underset{k}{⋃}C_{k}^{\left(L\right)}\right]\mathrm{dist}{\left(z_{i},C_{k_{i}^{*}}^{\left(L\right)}\right)}^{2},$$

where $\mathrm{dist}(z_{i},C_{k_{i}^{*}}^{\left(L\right)})$ denotes the distance from $z_{i}$ to the coverage region of its nearest leaf prototype. This term penalizes latent points outside all leaf coverage regions and pulls them toward the nearest predefined leaf region.

We further introduce a parent–child consistency loss to preserve the hierarchical decoding relation between connected prototypes. For a child prototype $p_{k}^{\left(\ell \right)}$ and its parent $p_{\pi_{\ell }\left(k\right)}^{\left(\ell -1\right)}$, the decoder is encouraged to reconstruct the child semantic target from the parent prototype and the parent-to-child coordinate residual:

(53) $$L_{\mathrm{pc}}=\sum_{\ell =1}^{L}\sum_{k\in I^{\left(\ell \right)}}{∥D_{\phi }\left(p_{\pi_{\ell }\left(k\right)}^{\left(\ell -1\right)},p_{k}^{\left(\ell \right)}-p_{\pi_{\ell }\left(k\right)}^{\left(\ell -1\right)}\right)-{\bar{x}}_{k}^{\left(\ell \right)}∥}_{2}^{2}.$$

This loss encourages the decoder to interpret a parent-to-child displacement as a semantic refinement from a coarser prototype to a finer prototype.

The main structural objective is formulated as follows.

(54) $$\begin{array}{cc} L_{\mathrm{struct}}= & \lambda_{\mathrm{rec}}L_{\mathrm{rec}}+\lambda_{\mathrm{proto}}L_{\mathrm{proto}}+\lambda_{\mathrm{mmd}}L_{\mathrm{mmd}}\\ \; & +\lambda_{\mathrm{cov}}L_{\mathrm{cov}}+\lambda_{\mathrm{pc}}L_{\mathrm{pc}}. \end{array}$$

With the auxiliary critic, the final autoencoder objective additionally includes the image-level refinement term:

(55) $$L_{\mathrm{total}}=L_{\mathrm{struct}}+\lambda_{\mathrm{ref}}L_{\mathrm{ref}}.$$

The critic itself is optimized separately using $L_{C}$. The coefficients $\lambda_{\mathrm{rec}}$, $\lambda_{\mathrm{proto}}$, $\lambda_{\mathrm{mmd}}$, $\lambda_{\mathrm{cov}}$, $\lambda_{\mathrm{pc}}$, and $\lambda_{\mathrm{ref}}$ control the relative importance of each term. In summary, the reconstruction loss preserves instance-level information, the prototype semantic loss encourages each fixed prototype to become interpretable as a representative visual anchor, and the MMD loss encourages latent embeddings to spread over the hyperbolic prototype scaffold. The coverage pullback loss improves the association between latent points and leaf coverage regions, while the parent–child consistency loss reinforces hierarchical decoding relations. The auxiliary refinement loss improves image sharpness without directly defining the prototype hierarchy.

**Figure 2:**
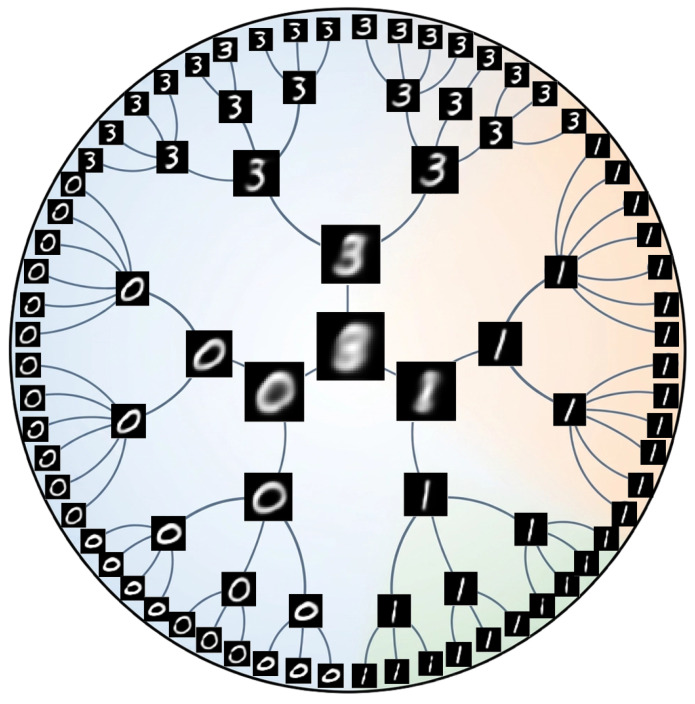
Conceptual illustration of the latent organization induced by the proposed method. The numeral images 0, 1, and 3 are illustrative prototype and instance-level decodings rather than class labels used during training. The root prototype represents a coarse shared semantic pattern, while prototypes at deeper levels decode into progressively more specific patterns along the fixed hyperbolic hierarchy. The connecting lines indicate parent–child relations between prototypes. The multiple numeral images around the leaf prototypes illustrate instance-level variations generated from local residual variations within each leaf region. The background colors are included only for visual clarity and carry no semantic or quantitative meaning.

Figure 2 conceptually illustrates the type of latent organization encouraged by the proposed method. Through prototype semantic learning, prototypes at shallow depths are expected to decode to coarse shared patterns, whereas deeper prototypes become progressively more specific. At the same time, MMD-based latent spreading encourages latent embeddings to occupy outer regions of the hyperbolic space, and the prototype-residual decoder models local instance-level variations around each leaf prototype. As a result, the latent space is intended to exhibit a coarse-to-fine hierarchical structure together with diverse local variations within each leaf region.

## 5. Experimental Setup

### 5.1. Overview of Experimental Settings

We evaluated the proposed method on two datasets with different levels of visual complexity: MNIST [29] and CelebA [30]. MNIST is used as a controlled setting to examine whether the proposed hyperbolic prototype-residual structure can form an interpretable latent organization, while CelebA is used to evaluate the behavior of the model on more complex natural images with richer appearance variations.

In all experiments, the latent space is modeled as a Poincaré ball with curvature −1, and the prototype coordinates are fixed during training. Since MNIST and CelebA differ substantially in image complexity, resolution, and training requirements, the preprocessing procedure, latent dimensionality, network structure, prototype tree configuration, loss weights, optimizer settings, batch size, and number of training epochs are specified separately for each dataset in the following subsections.

All experiments were implemented in PyTorch 2.0.1 with CUDA 11.7 and conducted on a workstation equipped with five NVIDIA RTX A5000 GPUs. For computationally demanding experiments, distributed training was used to accelerate model optimization.

### 5.2. MNIST Setup

MNIST is a standard handwritten-digit dataset consisting of ten digit classes from 0 to 9. We first conducted experiments on MNIST because it provides a relatively simple visual domain for examining the structural behavior of the proposed method. Each sample is a grayscale image 28×28, represented as $x\in R^{1\times 28\times 28}$. Apart from using the original image resolution and converting images to tensor format, no additional dataset-specific preprocessing is applied.

For MNIST, the latent dimension is set to d=3, and the latent space is modeled as the Poincaré ball with curvature −1. Since MNIST has a relatively simple visual structure, the prototype tree configuration is specified heuristically rather than being extensively searched. Specifically, we use a three-level branching configuration of 4–5–5, with level-wise radial steps with level-wise radial steps (0.6,0.4,0.3). The first-level prototypes are arranged from the origin using a simplex-based directional structure; in three dimensions, this yields four initial directions with a pairwise angular separation of approximately 109.47°. For deeper levels, each parent prototype generates five child prototypes using a child rotation angle of 50° from the outward direction of the parent, while spreading the children over the local tangent directions. Branching factors, radial steps, and angular settings are therefore used as heuristic structural parameters to analyze the proposed prototype hierarchy on MNIST.

The encoder maps an input image 1×28×28 to a latent vector in the tangent space, which is then mapped to the Poincaré ball using the exponential map at the origin. The decoder receives the concatenated prototype-residual representation and reconstructs a 1×28×28 grayscale image. The detailed network architecture is provided in Appendix A.

The model is trained for 30 epochs using Riemannian Adam with a model learning rate of $5\times 10^{-4}$. The structural loss weights are set to

$$\lambda_{\mathrm{rec}}=20.0,\;\lambda_{\mathrm{proto}}=5.0,\;\lambda_{\mathrm{mmd}}=1.0.$$

The auxiliary critic is introduced after three epochs and optimized with Adam using a learning rate of $1\times 10^{-4}$. Since the critic is used as an image-level refinement signal rather than as a source of latent hierarchy, its refinement weight is set to a relatively small value, $\lambda_{\mathrm{ref}}=0.25$, compared with the main structural loss weights. The resulting model is used to inspect prototype assignment and decoding, latent organization, and residual-based reconstruction in a relatively simple visual domain.

### 5.3. CelebA Setup

CelebA is a large-scale face image dataset containing diverse facial appearances, including variations in identity, hairstyle, pose, expression, and local texture. After the MNIST experiments, we evaluate the proposed method on CelebA to examine whether the hyperbolic prototype-residual structure can be applied to more complex natural images. Unlike MNIST, CelebA consists of RGB images with three channels. All images are resized to 64×64, and each sample is represented as $x\in R^{3\times 64\times 64}$.

For CelebA, the latent dimension is increased to d=32 to accommodate richer visual variations. As in the MNIST experiments, the latent space is modeled as the Poincaré ball with curvature −1. The prototype tree is initialized using a simplex-based root arrangement. Since a simplex structure in *d*-dimensional space produces d+1 initial directions, the first prototype level contains 33 prototypes. We then use a child branching configuration of 20–20, resulting in a three-level prototype hierarchy of 33–20–20.

While the prototype tree was specified heuristically for MNIST, the CelebA prototype placement is selected through an experimental search that balances coverage and overlap. Based on this search, we set the level-wise radial steps to 0.167, 0.270, and 0.220, with child rotation angles of 72° and 65° for the second and third prototype levels, respectively. The detailed search procedure for these placement parameters is provided in Appendix B.

The encoder and decoder follow the 64×64 architecture described in Appendix A. Briefly, the encoder maps an RGB image to a tangent-space latent vector through convolutional residual blocks, bottleneck self-attention, global pooling, and an MLP, followed by the exponential map at the origin. The decoder receives the prototype-residual representation as input, processes the prototype and residual through separate branches, and reconstructs a 64×64 RGB image using modulation, residual blocks, attention, and upsampling layers. This architecture is adapted to handle the increased visual complexity of natural face images.

The model is trained for approximately 400 epochs on CelebA. We use Riemannian Adam for the autoencoder with a model learning rate of $2.5\times 10^{-4}$, selective weight decay of $5\times 10^{-7}$, and a cosine learning-rate scheduler. The structural loss weights are set to

$$\lambda_{\mathrm{rec}}=50.0,\;\lambda_{\mathrm{proto}}=1.0,\;\lambda_{\mathrm{mmd}}=2.0.$$

Because CelebA contains richer image-level details than MNIST, the reconstruction weight is increased to better preserve visual details in natural face images, while the prototype and MMD weights are adjusted to maintain the hyperbolic prototype organization. The auxiliary critic is introduced after 10 epochs and optimized with Adam using a learning rate of $1\times 10^{-4}$. Since the critic is used primarily as an image-level refinement signal, its refinement weight is set to $\lambda_{\mathrm{ref}}=0.1$, which is smaller than the main structural loss weights. The resulting model is used to analyze reconstruction quality, prototype decoding, latent organization, and prototype-guided generation on complex face images.

The dataset-specific experimental configurations are summarized in Table 1.

## 6. Results

### 6.1. Results on MNIST

We first analyze the MNIST results to examine whether the proposed model forms the intended hyperbolic latent organization. Since MNIST has a relatively simple visual structure, it provides a suitable setting for visually inspecting latent spreading, prototype-based decoding behavior, and radial generation from the origin.

**Figure 3:**
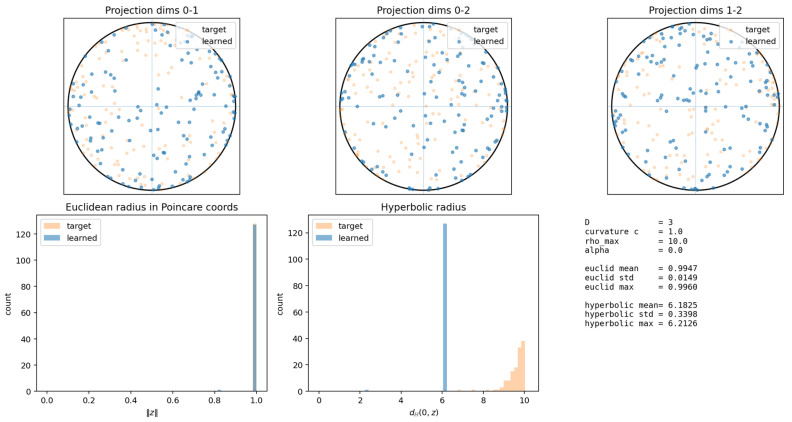
MMD-based latent spread on MNIST. The top row shows two-dimensional projections of target samples and encoded latent samples in the Poincaré ball. The bottom row shows Euclidean and hyperbolic radius histograms. The learned embeddings are pushed toward the outer region of the ball, indicating that MMD regularization prevents collapse near the origin and encourages radial spreading.

Figure 3 shows the distribution of encoded latent samples and target samples used for MMD regularization. The projected views indicate that the learned embeddings are not collapsed near the origin, but are instead distributed broadly across the Poincaré ball. The radial histograms further show that the encoded samples are concentrated near the outer region of the ball, which is consistent with the role of the MMD regularizer in encouraging outward latent spreading. Although the learned distribution does not exactly reproduce the target radial density, the result confirms that MMD regularization effectively prevents latent collapse and pushes the embeddings toward the peripheral region where the fixed prototype hierarchy is arranged.

**Figure 4:**
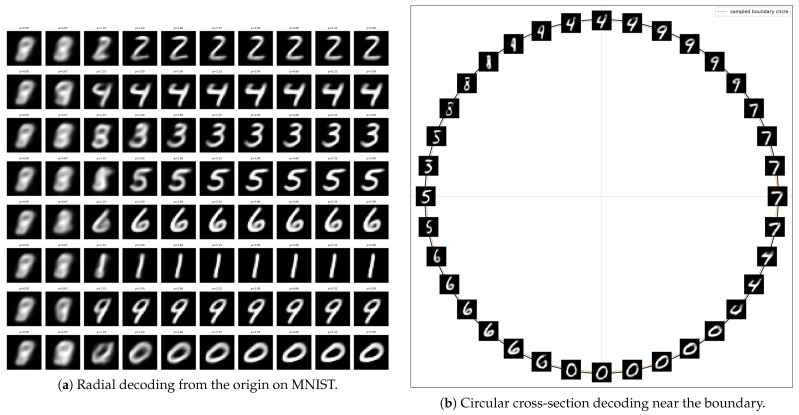
Qualitative decoding results on MNIST. (**a**) Radial decoding from the origin shows that decoded images evolve from blurred coarse patterns near the origin to sharper digit-specific samples near the boundary. (**b**) Circular cross-section decoding near the outer shell shows that different angular regions produce different digit-like samples, indicating that the boundary region contains multiple semantically distinct decoding regions.

Figure 4a presents decoded samples obtained by moving along selected radial directions from the origin. Near the origin, the decoded images are blurred and ambiguous, reflecting coarse visual information. As the latent point moves outward along each radial direction, the decoded images gradually become sharper and converge to digit-specific structures. This result supports the intended interpretation of the hyperbolic latent space: the central region represents shared coarse appearance, whereas points closer to the boundary encode more specific visual patterns. The fact that different radial directions lead to different digit identities also suggests that the angular component of the latent space captures semantic variation, while the radial component controls the degree of visual specificity.

To further inspect the behavior near the boundary, Figure 4b shows a circular cross-section decoding result in the Poincaré ball. Latent points are sampled along a circular trajectory near the outer shell, and each point is decoded through the prototype-residual decoder. The decoded images change according to the angular position on the circle, producing different digit-like samples along the boundary. This result indicates that the outer region of the latent space is not occupied by a single collapsed mode, but contains multiple semantically distinguishable regions. It also shows that the decoder can produce meaningful samples from latent points located near the boundary, supporting the use of leaf-level coverage regions for prototype-guided generation.

Overall, the MNIST results demonstrate that the proposed model forms a meaningful radial and angular organization in the hyperbolic latent space. MMD regularization spreads the embeddings toward the outer region, radial decoding reveals a transition from coarse to digit-specific reconstructions, and boundary sampling shows that different angular regions correspond to distinct digit-like outputs. These observations support the proposed prototype-residual interpretation, where the hyperbolic prototype tree provides global semantic structure and the residual component captures local variation around prototype regions.

To quantitatively examine the semantic organization of the prototype tree, each MNIST test sample was encoded and assigned to its nearest leaf prototype according to the hyperbolic distance. Its assignments at the preceding depths were subsequently recovered by following the corresponding parent nodes. Class labels were not used during this assignment process and were introduced only for post-hoc evaluation.

**Figure 5:**
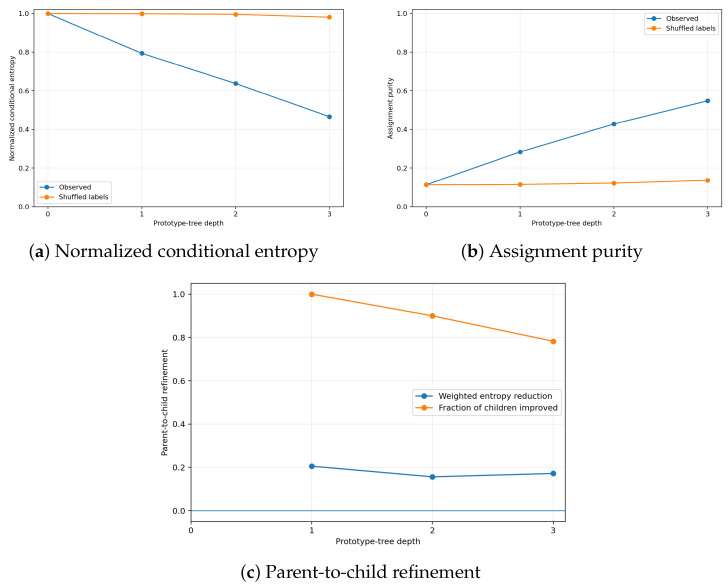
Quantitative evaluation of semantic organization across the MNIST prototype tree. (**a**) Normalized conditional entropy of the observed prototype assignments and the shuffled-label reference. (**b**) Assignment purity at each prototype-tree depth. (**c**) Parent-to-child refinement, evaluated using the weighted entropy reduction and the fraction of child nodes exhibiting lower entropy than their corresponding parents. With increasing depth, the observed assignments become more class-specific, whereas the shuffled-label reference exhibits little depth-dependent change.

Figure 5 compares the resulting assignments with a shuffled-label reference. The normalized conditional entropy decreased from approximately 1.00 at the root to 0.79, 0.64, and 0.47 at successive depths, whereas the shuffled-label values remained close to 1.00. In parallel, assignment purity increased from approximately 0.11 to 0.28, 0.43, and 0.55, while the shuffled reference remained between 0.11 and 0.14. These results show that deeper prototype nodes contain progressively more class-specific sample groups rather than merely producing finer random partitions.

The parent-to-child analysis provides a complementary examination of the tree relations. The weighted entropy reductions were approximately 0.20, 0.15, and 0.17 across the three successive depth transitions, and the fractions of child nodes exhibiting lower entropy than their respective parents were 1.00, 0.90, and 0.78. Accordingly, most child nodes refine the label composition of their parents as the tree becomes deeper. These observations support progressive semantic specialization along the prototype tree, without assuming that the learned structure reproduces a predefined class taxonomy. Detailed definitions of the metrics, the shuffled-label procedure, and the complete depth-wise statistics are provided in Appendix D.

### 6.2. Results on CelebA

We next examine the results on CelebA to evaluate whether the proposed hyperbolic prototype-residual structure remains meaningful on a more complex natural-image domain. Compared with MNIST, CelebA contains substantially richer appearance variations, including hairstyle, facial shape, pose, illumination, and background tone. The qualitative results therefore focus on whether the model can organize such variations into interpretable prototype-level semantics, radial coarse-to-fine decoding behavior, and local variations around leaf regions.

**Figure 6:**
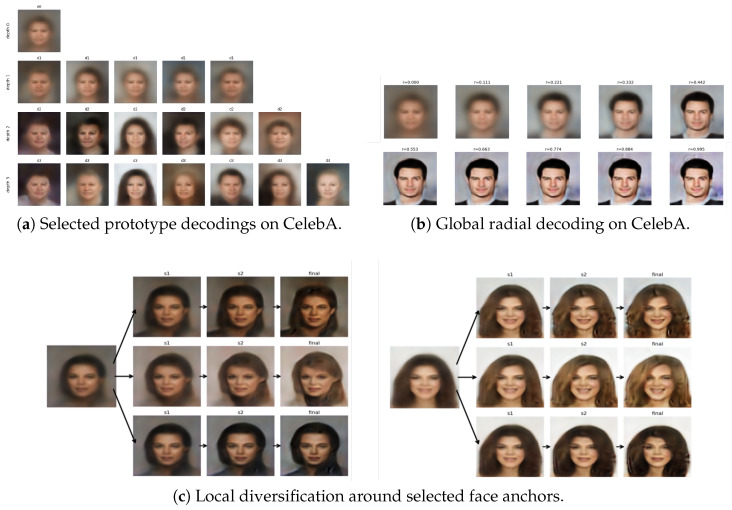
Qualitative analysis of prototype-guided latent organization on CelebA. (**a**) Selected prototype decodings across different depths of the fixed hyperbolic prototype tree. The root prototype produces a coarse average face, while deeper prototypes become progressively more specific and semantically differentiated. (**b**) Global radial decoding along selected leaf directions. Each row corresponds to one selected radial direction, and each column shows the decoded image at an increasing radial coordinate from the origin to the outer region of the Poincaré ball. The decoded outputs evolve from blurred coarse faces to sharper and more specific face-like samples. (**c**) Controlled local diversification under a fixed prototype. Within each group, the same prototype anchor is retained while the residual is varied. The generated images preserve a shared prototype-level facial structure while exhibiting local instance-level differences.

Figure 6a shows selected prototype decodings across different depths of the fixed hyperbolic prototype tree. The root prototype produces a coarse average face, reflecting the most globally shared visual structure of the dataset. As the depth increases, the decoded prototypes become progressively more specific, revealing more localized facial tendencies such as hairstyle, face shape, tone, and background appearance. These results suggest that the prototype semantic learning objective successfully assigns interpretable visual meaning to the fixed prototype coordinates. In other words, the prototypes are not merely geometric anchors in the latent space, but function as representative semantic reference points for different face regions.

Figure 6b visualizes decoded images along selected radial directions from the origin toward the boundary of the Poincaré ball. In all directions, the decoded image near the origin appears as a blurred and highly averaged face. As the radial coordinate increases, the decoded outputs gradually become sharper and more specific, eventually converging to distinct face-like samples near the outer region. This behavior is consistent with the intended interpretation of the latent space: the central region captures shared coarse appearance, whereas the peripheral region encodes more detailed and instance-specific structure. Moreover, different radial directions lead to different facial tendencies, indicating that angular location and radial depth jointly organize semantic variation in the learned latent space.

Figure 6c shows controlled local diversification under fixed prototype anchors. Within each group, the prototype is held fixed while the residual is varied, producing several related but distinct face images. The generated samples preserve a shared prototype-level facial structure while differing in finer visual attributes such as hairstyle, facial contour, brightness, and local texture. This result supports the prototype-residual interpretation of the proposed model: the prototype provides a semantic anchor, whereas the residual captures instance-level variation within the corresponding prototype region.

To evaluate reconstruction and generative performance on CelebA, we compared the proposed model with VAE and WAE baselines under a common evaluation protocol. Reconstruction quality was measured using mean squared error. For generative evaluation, each model independently generated 10,000 random images using its corresponding latent sampling procedure. These generated images, rather than reconstructed images, were compared with the same set of 2599 held-out real CelebA images. FID and KID were computed using Inception-v3 features, while the real-image set, generated sample count, image preprocessing, and metric implementation were kept fixed across all models. Table 2 reports the mean and standard deviation over three independent runs.

The WAE achieved the lowest reconstruction MSE, demonstrating the best pixel-level reconstruction among the compared models. The proposed model obtained the lowest FID and KID, suggesting closer agreement between the generated and real-image distributions. The VAE performed worst across all three metrics. Overall, the proposed model provided the strongest distribution-level generative performance, whereas the WAE retained an advantage in reconstruction fidelity.

Overall, the CelebA results indicate that the proposed model organizes the latent space in a coarse-to-fine manner that is visually interpretable. Prototype decoding reveals meaningful hierarchical face anchors, radial decoding shows a transition from shared coarse appearance to detailed face generation, and local branching around leaf regions demonstrates that diverse instance-level variations can emerge while preserving prototype-level semantics.

### 6.3. Ablation Study

#### 6.3.1. Effect of MMD-Based Latent Spreading

**Figure 7:**
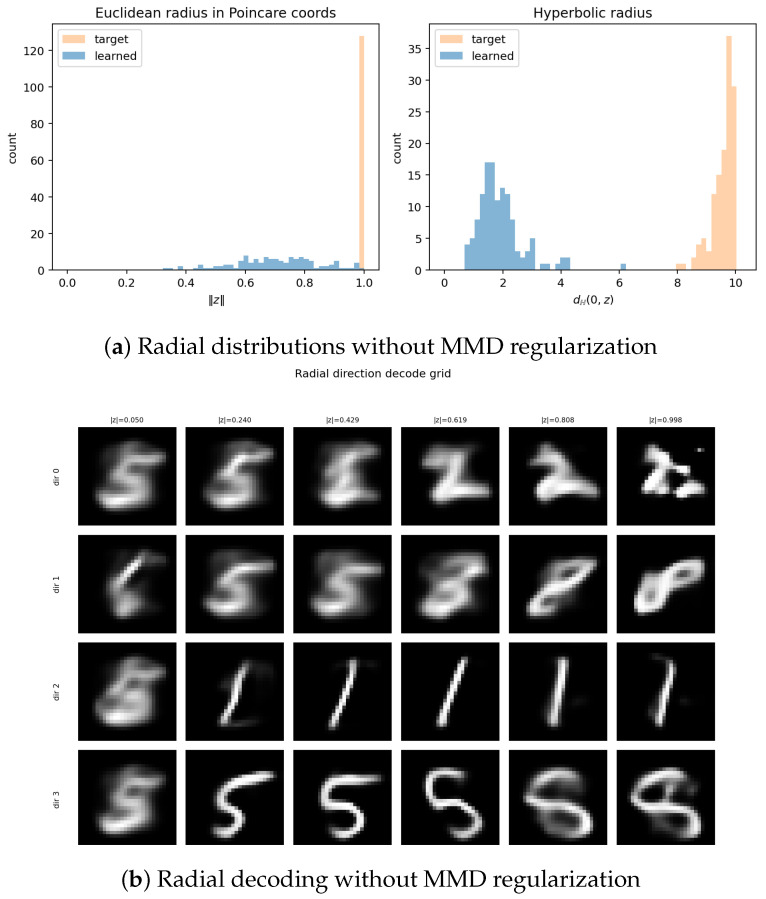
Effect of removing the MMD-based latent spreading objective on MNIST. (**a**) Euclidean and hyperbolic radius distributions of the hyperbolic volume-aware target samples and the encoded samples obtained without MMD regularization. The learned embeddings are broadly distributed over intermediate radii and fail to match the target distribution concentrated near the peripheral region. (**b**) Radial decoding results of the model trained without MMD. Each row corresponds to a fixed latent direction, and each column represents an increasing radius from the origin toward the boundary. Without MMD, several directions exhibit blurred, semantically inconsistent, or distorted outputs as the radius increases.

To examine the contribution of the MMD-based latent spreading objective, we trained an ablated model without $L_{\mathrm{mmd}}$, while keeping the remaining model architecture and training objectives unchanged. Figure 7 compares the resulting radial latent distribution and radial decoding behavior.

As shown in Figure 7a, removing the MMD objective causes the encoded latent distribution to deviate substantially from the hyperbolic volume-aware target distribution. While the target samples are concentrated near the peripheral region of the Poincaré ball, the learned embeddings are broadly dispersed over intermediate Euclidean and hyperbolic radii. In particular, the learned hyperbolic radii occupy a substantially narrower range than the target distribution and exhibit irregular gaps. Consequently, the radial position of an encoded point no longer follows the intended distribution from central coarse regions toward peripheral specific regions.

This mismatch also affects the decoding behavior along the radial direction. Figure 7b shows images decoded along fixed directions while progressively increasing the radius from the origin toward the boundary. Without MMD regularization, increasing the radius does not consistently produce a stable coarse-to-specific transition. Although some directions preserve recognizable digit structures, several directions exhibit blurred outputs, abrupt semantic changes, or severe distortions near the outer region. This behavior indicates that the decoder is not trained uniformly over the intended radial range when the encoded samples fail to occupy the volume-aware target regions.

These results suggest that the MMD objective does not merely prevent latent collapse. It aligns the aggregated encoded distribution with the intended hyperbolic radial structure, thereby providing training support over a broader and more geometrically meaningful range of the Poincaré ball. As a result, MMD regularization contributes to a more consistent and interpretable radial decoding behavior.

#### 6.3.2. Effect of the Image-Level Critic

**Figure 8:**
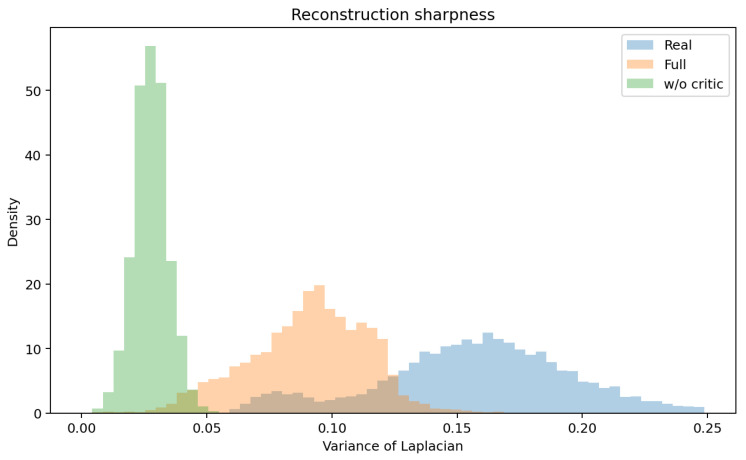
Distribution of reconstruction sharpness measured by the variance of the Laplacian. The full model exhibits a sharpness distribution closer to that of real MNIST images, whereas the model trained without the auxiliary critic is concentrated in a lower-sharpness region.

To examine the auxiliary effect of the image-level critic, we compared the full model with a variant trained without the critic using the variance of the Laplacian as a sharpness measure. As shown in Figure 8, the mean Laplacian variance of the reconstructed images was 0.0907 for the full model and 0.0276 without the critic, while the value for real MNIST images was 0.1582. A similar tendency was observed for generated samples, with values of 0.1384 and 0.0274, respectively. The resulting distributions indicate that the critic helps the reconstructed and generated images retain edge sharpness closer to that of the real images. These results support the use of the critic as an auxiliary image-level refinement component.

#### 6.3.3. Effect of the Coverage Pullback Loss

We further evaluated the effect of the coverage pullback loss $L_{\mathrm{cov}}$. On MNIST, removing this term produced little measurable difference, suggesting that the low-dimensional latent space already achieved sufficient coverage under the fixed prototype arrangement. In contrast, its effect became substantial on CelebA with a 32-dimensional latent space. Without $L_{\mathrm{cov}}$, 60.6% of the encoded samples were located outside the union of the predefined leaf coverage regions. Consequently, these samples were excluded from all leaf-level coverage-based semantic target sets. With the coverage pullback loss, the average coverage rate increased to 91.5%, meaning that most encoded samples were located within at least one leaf coverage region. These results show that $L_{\mathrm{cov}}$ substantially reduces the drift of high-dimensional embeddings into uncovered regions and maintains their association with the predefined prototype structure.

## 7. Discussion

The experimental results suggest that the proposed hyperbolic prototype-residual autoencoder provides an interpretable way to organize generative latent representations. The MMD-based latent regularization encourages encoded samples to occupy broad regions of the Poincaré ball, while radial decoding shows a coarse-to-specific transition from the origin toward the boundary. This behavior supports the intended geometric interpretation of the latent space, where the central region captures shared coarse appearance and the outer region represents more specific visual patterns.

A key observation is that fixed prototypes can acquire visual meaning through decoder training, even though their coordinates are not learned. The prototype semantic loss encourages each fixed prototype to decode into a representative visual anchor. As a result, the fixed hyperbolic prototype tree does not merely act as a geometric partition of the latent space, but also provides a semantic scaffold for organizing the decoding behavior of the model. This effect is especially visible in CelebA, where deeper prototypes correspond to more differentiated face-like appearances.

The prototype-residual decomposition also contributes to the interpretability of the generation process. Instead of decoding from a single latent vector, the decoder receives a selected prototype and a coordinate residual. The prototype provides a coarse semantic anchor, while the residual introduces local instance-level variation around that anchor. This decomposition allows the generated outputs to be analyzed in terms of both prototype-level anchoring and local variation within a prototype region. The radial decoding and leaf-level diversification results support this interpretation by showing that changes in radial position, angular direction, and local residual variation lead to different generated outputs.

The auxiliary critic further improves image-level sharpness, but it should not be interpreted as the source of the hyperbolic hierarchy. The hierarchy is primarily determined by the fixed prototype tree, prototype assignment, prototype semantic learning, MMD-based latent spreading, and structural regularizers. The critic primarily serves as a complementary image-level refinement signal for model-generated images, although its gradients may indirectly influence the learned latent representations. This distinction is important because the main contribution of the proposed method lies in latent organization and prototype-level interpretability rather than adversarial training itself.

Several limitations remain. First, the prototype tree configuration is manually specified or selected through empirical search and may not be optimal for datasets with complex or unknown semantic organization. Second, quantitative semantic-hierarchy validation was conducted only on MNIST, where mutually exclusive digit labels provide a controlled semantic reference. The CelebA results remain primarily qualitative because its flat, non-mutually-exclusive binary attributes do not define a ground-truth hierarchy and do not fully capture factors such as pose, illumination, background, texture, and continuous appearance variation. Accordingly, depth-wise attribute purity would measure alignment with selected annotations rather than provide an unambiguous evaluation of the learned hierarchy. Third, high-dimensional hyperbolic latent spaces remain difficult to visualize and must often be analyzed indirectly through decoded images and selected projections.

Future work may extend the proposed framework in several directions. One promising direction is to learn an explicit residual prior or a conditional residual distribution around each prototype. In the current formulation, generation is performed by selecting a prototype and sampling latent points within its coverage region, and the residual is used as a decoder-side local variation vector. Learning a conditional prior such as p(r∣p) could provide a more principled probabilistic sampling mechanism and improve the consistency of prototype-guided generation. Another direction is to make the prototype tree adaptive, allowing branching factors, prototype placement, or coverage regions to be learned from data rather than fixed in advance. Finally, scaling the framework to higher-resolution images would require stronger decoder architectures and more efficient sampling or training strategies.

Overall, the results indicate that a fixed hyperbolic prototype tree can serve as an interpretable scaffold for organizing generative latent spaces. By combining prototype-level semantic anchors with residual-based local variation, the proposed framework provides a structured way to analyze and control image reconstruction and generation.

## 8. Conclusions

In this paper, we proposed a hyperbolic prototype-residual autoencoder for interpretable generative latent organization. The main idea is to embed a fixed hierarchical prototype tree in the Poincaré ball and decompose each latent representation into a selected prototype and a coordinate residual. Through this design, image reconstruction and generation are organized around two complementary factors: prototype-level semantic anchoring and instance-level residual variation.

The proposed framework makes three main contributions. First, it introduces a fixed hyperbolic prototype scaffold that provides an explicit coarse-to-fine structure in the latent space. Unlike approaches that rely only on unconstrained continuous latent variables, the proposed method assigns each encoded sample to a prototype according to hyperbolic distance, allowing the latent space to be interpreted through a predefined hierarchical organization. Second, it introduces prototype semantic learning, which encourages fixed prototype coordinates to acquire visual meaning through decoder training. As a result, prototypes become representative visual anchors rather than merely geometric reference points. Third, it adopts a prototype-residual decoding mechanism, where the prototype specifies the global semantic anchor and the residual captures local instance-level variation around that anchor.

To support this structure, we used MMD-based latent regularization to encourage encoded samples to spread over the hyperbolic latent space, together with structural regularizers such as coverage pullback and parent-child consistency. An auxiliary critic was further employed as an image-level refinement module to improve the sharpness of model-generated images, without directly defining the hyperbolic hierarchy.

Experiments on MNIST and CelebA demonstrated that the proposed method forms an interpretable coarse-to-fine organization in the latent space. MMD regularization encouraged latent embeddings to spread toward the outer region of the Poincaré ball, radial decoding showed a transition from coarse averaged images near the origin to sharper and more specific images near the boundary, and prototype decoding showed that fixed prototypes can serve as meaningful semantic anchors. In particular, the CelebA results showed that deeper prototypes and leaf-level variations can represent differentiated face-like appearances while preserving prototype-level structure.

Overall, the results suggest that a fixed hyperbolic prototype tree can serve as an effective semantic scaffold for interpretable generative modeling. By separating prototype-level semantics from residual-level variation, the proposed method provides a structured way to analyze and control image reconstruction and generation. Future work may extend this framework by learning adaptive prototype trees, introducing explicit residual priors for prototype-conditioned sampling, and scaling the model to higher-resolution image generation.

## Figures and Tables

**Table 1:** Dataset-specific training configurations used for the proposed model. All values correspond to the final models used in the reported experiments.

Configuration	MNIST	CelebA
*Dataset and input *		
Training data	Standard MNIST training split: 50,000 images	Standard CelebA training split: 200,000 images
Input resolution	1×28×28	3×64×64
Preprocessing	No additional augmentation	Resize to 64×64
*Latent space and prototype tree*		
Latent dimension	d=3	d=32
Poincaré curvature	−1	−1
Prototype coordinates	Fixed during training	Fixed during training
Root arrangement	Simplex-based directions	Simplex-based directions
Branching configuration	4–5–5	33–20–20
Total number of prototypes	1+4+20+100=125	1+33+660+13,200=13,894
Level-wise radial steps	0.600,0.400,0.300	0.166812,0.270,0.220
Child rotation angles	50° at the deeper levels	72°,65°
Prototype assignment	Nearest leaf by hyperbolic distance	Nearest leaf by hyperbolic distance
*Model optimization*		
Batch size	1000	400
Training epochs	30	400
Autoencoder optimizer	Riemannian Adam	Riemannian Adam
Initial learning rate	$5.0\times 10^{-4}$	$2.5\times 10^{-4}$
Weight decay	0	$5.0\times 10^{-7}$
Learning-rate scheduler	None	Cosine scheduler
Scheduler parameters	None	Minimum learning rate: $1.0\times 10^{-6}$
*Structural objectives*		
Reconstruction weight	$\lambda_{\mathrm{rec}}=20.0$	$\lambda_{\mathrm{rec}}=50.0$
Prototype semantic weight	$\lambda_{\mathrm{proto}}=5.0$	$\lambda_{\mathrm{proto}}=1.0$
MMD weight	$\lambda_{\mathrm{mmd}}=1.0$	$\lambda_{\mathrm{mmd}}=2.0$
Coverage pullback weight	$\lambda_{\mathrm{cov}}=0.001$	$\lambda_{\mathrm{cov}}=0.15$
Parent–child weight	$\lambda_{\mathrm{pc}}=0.25$	$\lambda_{\mathrm{pc}}=0.2$
*Auxiliary critic*		
Critic start epoch	3	10
Refinement weight	$\lambda_{\mathrm{ref}}=0.25$	$\lambda_{\mathrm{ref}}=0.10$
Critic optimizer	Adam	Adam
Critic learning rate	$1.0\times 10^{-4}$	$1.0\times 10^{-4}$

**Table 2:** Quantitative comparison with VAE and WAE on CelebA. Results are reported as the mean ± standard deviation over three independent runs. The downward arrow (↓) indicates that lower values are better.

Model	Reconstruction MSE ↓	FID ↓	KID ↓
Proposed	0.013113±0.000222	74.0189±8.0055	0.074219±0.012100
VAE	0.018288±0.000414	123.9151±2.2049	0.111202±0.002118
WAE	0.011314±0.000058	98.5120±5.5699	0.098548±0.006764

Note: Bold values indicate the best performance for each metric.

**Figure A1:**
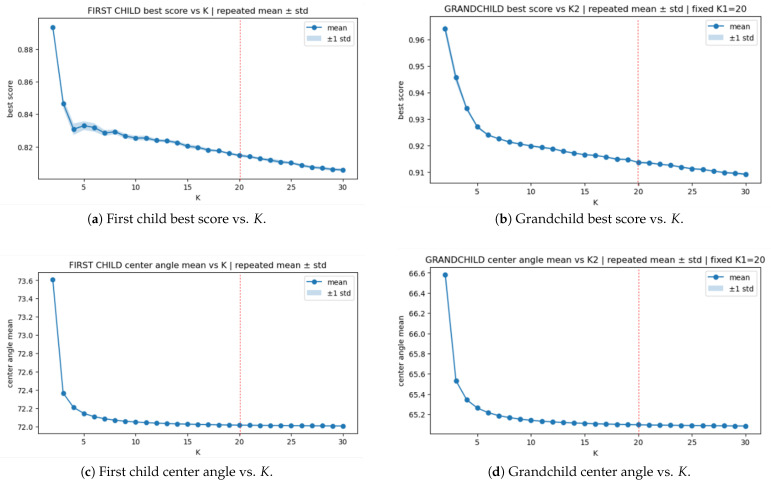
Repeated branching-factor and angle search for the CelebA prototype tree. (**a**,**b**) show the best full-shell score as a function of the number of child nodes per parent *K* for the first child level and the grandchild level, respectively. (**c**,**d**) show the corresponding mean center angle, which measures the average angular deviation of child prototype directions from the outward direction of their parent prototype. The shaded regions indicate one standard deviation over repeated runs. The red dashed vertical lines indicate the selected branching factor K=20 used in the final prototype-tree configuration. Based on this search, we use the branching configuration (20,20) and approximate the optimized child patterns using rotation angles of approximately 72° and 65° for the child and grandchild levels, respectively.

**Figure A2:**
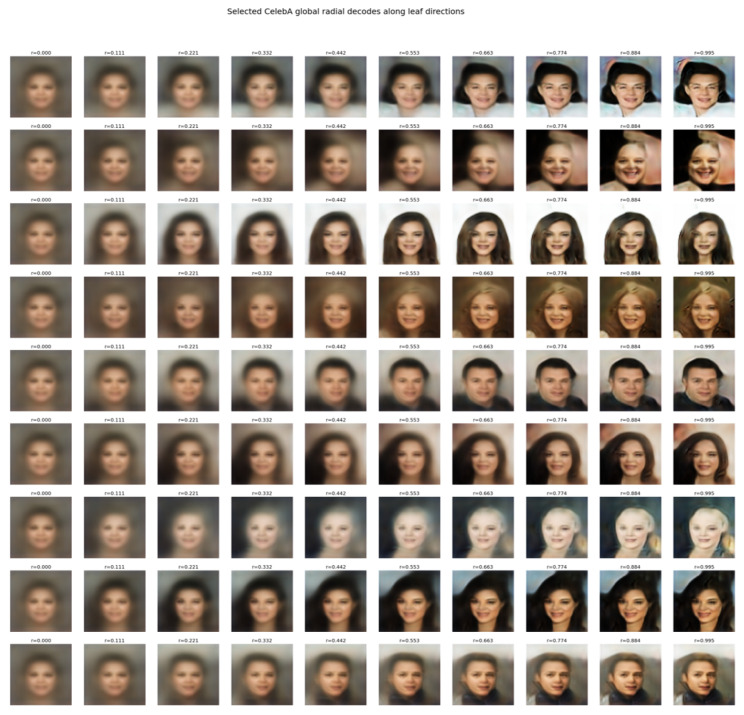
Examples of local diversification around selected face anchors on CelebA. Starting from a common coarse face, nearby latent variations lead to multiple related but distinct outputs, illustrating that leaf-level regions can support diverse instance-level generations while preserving coarse semantic structure.

## Data Availability

Publicly available datasets were analyzed in this study. The MNIST dataset can be found at http://yann.lecun.com/exdb/mnist/ (accessed on 22 July 2026), and the CelebA dataset is available at http://mmlab.ie.cuhk.edu.hk/projects/CelebA.html (accessed on 22 July 2026).

## Appendix A. Network Architecture

This appendix summarizes the dataset-specific network architectures used in the experiments. Both MNIST and CelebA use the same hyperbolic prototype-residual autoencoder framework, but the encoder and decoder architectures are adjusted according to the image resolution and visual complexity of each dataset.

**Table A1:** Basic neural network blocks used in the encoder, decoder, and critic.

Block	Operation	Description
ResBlock	$\mathrm{GN}\to \mathrm{SiLU}\to {\mathrm{Conv}}_{3\times 3}\to \mathrm{GN}\to \mathrm{SiLU}\to {\mathrm{Conv}}_{3\times 3}$	Residual convolutional block with a 1×1 skip projection when the channel dimension or stride changes.
UpBlock	Bilinear upsampling ×2, followed by two 3×3 convolutional layers with normalization and SiLU activation	Upsampling block used in the decoder to increase spatial resolution. A 1×1 skip projection is applied after upsampling.
SelfAttention2d	GroupNorm, 1×1 QKV projection, multi-head scaled dot-product attention, 1×1 output projection	Bottleneck attention module applied at low spatial resolution.

**Table A2:** MNIST encoder architecture.

Module	Layer/Operation	Output Size
Input	–	1×28×28
stem	Conv2d(1,32,3,1,1)	32×28×28
block1	ResBlock(32,32,stride=1)	32×28×28
block2	ResBlock(32,64,stride=2)	64×14×14
block3	ResBlock(64,64,stride=1)	64×14×14
block4	ResBlock(64,128,stride=2)	128×7×7
block5	ResBlock(128,128,stride=1)	128×7×7
trunk_head	Flatten→Linear(128·7·7,256)→SiLU→Linear(256,128)→SiLU	128
latent_head	Linear(128,d)	*d*
Hyperbolic map	${\mathrm{Exp}}_{0}^{c}\left(u_{e}\right)$, followed by projection	$z_{e}\in B_{c}^{d}$

**Table A3:** MNIST decoder architecture.

Module	Layer/Operation	Output Size
Input	$\left[p_{\mathrm{anchor}},r\right]$	2d
fc	$\begin{array}{c} \mathrm{Linear}(2d,128)\to \mathrm{SiLU}\\ \;\to \mathrm{Linear}(128,256)\to \mathrm{SiLU}\\ \;\to \mathrm{Linear}(256,128\times 7\times 7)\to \mathrm{SiLU} \end{array}$	128×7×7
Reshape	–	128×7×7
block1	ResBlock(128,128,stride=1)	128×7×7
up1	UpBlock(128,64)	64×14×14
block2	ResBlock(64,64,stride=1)	64×14×14
up2	UpBlock(64,32)	32×28×28
block3	ResBlock(32,32,stride=1)	32×28×28
out	$\begin{array}{c} \mathrm{GN}\to \mathrm{SiLU}\to \mathrm{Conv}2d(32,1,3,1,1)\to \mathrm{Sigmoid} \end{array}$	1×28×28

**Table A4:** CelebA encoder architecture.

Module	Layer/Operation	Output Size
Input	–	3×64×64
stem	Conv2d(3,48,3,1,1)	48×64×64
enc1	$\begin{array}{c} \mathrm{ResBlock}(48,48,\mathrm{stride}=1)\\ \;\to \mathrm{ResBlock}(48,48,\mathrm{stride}=1) \end{array}$	48×64×64
enc2	$\begin{array}{c} \mathrm{ResBlock}(48,96,\mathrm{stride}=2)\\ \;\to \mathrm{ResBlock}(96,96,\mathrm{stride}=1) \end{array}$	96×32×32
enc3	$\begin{array}{c} \mathrm{ResBlock}(96,160,\mathrm{stride}=2)\\ \;\to \mathrm{ResBlock}(160,160,\mathrm{stride}=1) \end{array}$	160×16×16
enc4	$\begin{array}{c} \mathrm{ResBlock}(160,224,\mathrm{stride}=2)\\ \;\to \mathrm{ResBlock}(224,224,\mathrm{stride}=1) \end{array}$	224×8×8
mid_attn	SelfAttention2d(224) or identity	224×8×8
pool	AdaptiveAvgPool2d(1)	224×1×1
trunk_head	$\begin{array}{c} \mathrm{Flatten}\to \mathrm{Linear}(224,256)\to \mathrm{SiLU}\\ \;\to \mathrm{Linear}(256,128)\to \mathrm{SiLU} \end{array}$	128
latent_head	Linear(128,d)	*d*
Hyperbolic map	${\mathrm{Exp}}_{0}^{c}\left(u_{e}\right)$, followed by projection	$z_{e}\in B_{c}^{d}$

**Table A5:** CelebA decoder architecture.

Module	Layer/Operation	Output Size
Prototype input	*p*	*d*
Residual input	*r*	*d*
proto_mlp	$\begin{array}{c} \mathrm{Linear}(d,192)\to \mathrm{SiLU}\\ \;\to \mathrm{Linear}(192,384)\to \mathrm{SiLU} \end{array}$	384
residual_mlp	$\begin{array}{c} \mathrm{Linear}(d,192)\to \mathrm{SiLU}\\ \;\to \mathrm{Linear}(192,384)\to \mathrm{SiLU} \end{array}$	384
film	Linear(384,768)	$\left(\gamma ,\beta \right)\in R^{384}\times R^{384}$
Feature fusion	$h=r_{\mathrm{feat}}⊙\left(1+0.1\tanh\left(\gamma \right)\right)+\beta$	384
fc	Linear(384,192·8·8)→SiLU	192×8×8
dec0	$\begin{array}{c} \mathrm{ResBlock}(192,192,\mathrm{stride}=1)\\ \;\to \mathrm{ResBlock}(192,192,\mathrm{stride}=1) \end{array}$	192×8×8
mid_attn	SelfAttention2d(192) or identity	192×8×8
up1	UpBlock(192,160)	160×16×16
dec1	$\begin{array}{c} \mathrm{ResBlock}(160,160,\mathrm{stride}=1)\\ \;\to \mathrm{ResBlock}(160,160,\mathrm{stride}=1) \end{array}$	160×16×16
up2	UpBlock(160,96)	96×32×32
dec2	$\begin{array}{c} \mathrm{ResBlock}(96,96,\mathrm{stride}=1)\\ \;\to \mathrm{ResBlock}(96,96,\mathrm{stride}=1) \end{array}$	96×32×32
up3	UpBlock(96,48)	48×64×64
dec3	$\begin{array}{c} \mathrm{ResBlock}(48,48,\mathrm{stride}=1)\\ \;\to \mathrm{ResBlock}(48,48,\mathrm{stride}=1) \end{array}$	48×64×64
out	$\begin{array}{c} \mathrm{GN}\to \mathrm{SiLU}\\ \;\to \mathrm{Conv}2d(48,3,3,1,1)\\ \;\to \mathrm{Sigmoid} \end{array}$	3×64×64

## Appendix B. Prototype Placement Search

To determine the fixed prototype tree used for CelebA, we performed a geometric placement search prior to model training. The search was conducted in the same latent dimension as the CelebA model, d=32, using a simplex-based initial arrangement. Since a simplex in *d*-dimensional space produces d+1 directions, this gives 33 initial prototype directions.

We first selected the branching factors by evaluating candidate values of the number of child nodes per parent. For each candidate *K*, we measured a full-shell score that balances uncovered and overlapping regions of the latent shell:

(A1) $$S=R_{\mathrm{uncov}}+R_{\mathrm{overlap}},$$

where $R_{\mathrm{uncov}}$ denotes the fraction of shell samples not covered by any prototype region, and $R_{\mathrm{overlap}}$ denotes the fraction of shell samples covered by two or more prototype regions. Lower scores therefore indicate that the prototype regions provide broader coverage while avoiding excessive overlap.

As shown in Figure A1, the score decreases rapidly when *K* increases from small values and then enters a low-score saturated regime. We therefore select K=20 for the first child level. After fixing the first branching factor to 20, we repeated the same search for the grandchild level and again selected K=20. This gives the final CelebA branching configuration (20,20). This choice should be interpreted as a stable configuration in the saturated low-score regime rather than as a unique optimum.

We also estimated the angular placement of child prototypes from the optimized child patterns. The center angle measures the average angular deviation of child prototype directions from the outward direction of their parent prototype. At the selected branching factor K=20, the mean center angles are approximately 72° for the first child level and 65° for the grandchild level. These values are used as single-angle approximations of the optimized child patterns in the final prototype-tree implementation.

The selected prototype tree is fixed before training. During training, the encoder and decoder are optimized to align latent representations and generated images with this pre-arranged hyperbolic prototype hierarchy.

## Appendix D. Details of the Semantic-Hierarchy Evaluation

### Appendix D.1. Prototype Assignment Procedure

Let $z_{i}$ denote the encoded representation of the *i*-th test sample and let $P^{L}$ denote the set of leaf prototypes at the deepest tree level *L*. The leaf assignment was determined using the nearest prototype under the hyperbolic distance:

(A2) $$a_{i}^{\left(L\right)}=\arg\min_{p\in P_{L}}d_{B_{c}}\left(z_{i},p\right).$$

After assigning a sample to a leaf prototype, its assignments at the shallower depths were obtained by tracing the stored parent indices from the selected leaf to the root. Consequently, each sample followed a single path through the fixed prototype tree. Ground-truth labels were not involved in prototype selection and were used only to evaluate the semantic composition of the resulting nodes.

### Appendix D.2. Depth-Wise Entropy and Purity

For a node *j* at depth *d*, let $n_{d,j,k}$ be the number of assigned samples belonging to class *k*, and let

(A3) $$n_{d,j}=\sum_{k=1}^{K}n_{d,j,k},\;q_{d,j,k}=\frac{n_{d,j,k}}{n_{d,j}},$$

where *K* is the number of classes. The normalized entropy of node *j* is defined as

(A4) $$H_{d,j}=-\frac{1}{\log K}\sum_{k=1}^{K}q_{d,j,k}\log q_{d,j,k}.$$

The depth-wise normalized conditional entropy is obtained by weighting each node according to its number of assigned samples:

(A5) $$H_{d}=\sum_{j}\frac{n_{d,j}}{N}H_{d,j},$$

where *N* is the total number of evaluated samples. Lower values indicate that the nodes at the corresponding depth contain more class-specific assignments.

The assignment purity at depth *d* is defined as

(A6) $$P_{d}=\sum_{j}\frac{n_{d,j}}{N}\max_{k}q_{d,j,k}.$$

Higher purity indicates that a larger proportion of samples agrees with the majority class of its assigned prototype node.

### Appendix D.3. Shuffled-Label Reference

To distinguish semantic organization from changes caused only by an increasing number of prototype nodes, a shuffled-label reference was constructed by randomly permuting the class labels while preserving all prototype assignments. The entropy and purity were then recomputed using the permuted labels. Because the assignment structure remains unchanged, differences between the observed and shuffled results reflect the association between the learned prototype partition and the original class labels.

### Appendix D.4. Parent-to-Child Refinement

For a non-empty child node *c* and its parent π(c), semantic refinement was evaluated by comparing their normalized entropies. The weighted entropy reduction for the transition from depth d−1 to depth *d* is defined as

(A7) $$\Delta H_{d}=\sum_{c\in V_{d}^{+}}\frac{n_{c}}{N}\left[H_{\pi (c)}-H_{c}\right],$$

where $V_{d}^{+}$ denotes the set of non-empty child nodes at depth *d*. A positive value indicates that child nodes have lower label uncertainty than their parents on average.

The fraction of improved child nodes is given by

(A8) $$R_{d}=\frac{1}{|V_{d}^{+}|}\sum_{c\in V_{d}^{+}}I\left[H_{c}<H_{\pi (c)}\right].$$

This measure reports how consistently the reduction occurs across individual parent-to-child relations rather than only in the depth-wise average.

**Table A6:** Depth-wise semantic-organization metrics for the MNIST prototype assignments. The values are rounded to two decimal places.

Depth	Observed Entropy ↓	Shuffled Entropy	Observed Purity ↑	Shuffled Purity
0	1.00	1.00	0.11	0.11
1	0.79	1.00	0.28	0.11
2	0.64	1.00	0.43	0.12
3	0.47	0.98	0.55	0.14

Note: The downward arrow (↓) indicates that lower values are better, whereas the upward arrow (↑) indicates that higher values are better.

**Table A7:** Parent-to-child semantic refinement across successive prototype-tree depth transitions.

Depth Transition	Weighted Entropy Reduction ↑	Fraction of Children Improved ↑
0→1	0.20	1.00
1→2	0.15	0.90
2→3	0.17	0.78

Note: The upward arrow (↑) indicates that higher values are better.
